# Single-molecule characterization of extrinsic transcription termination by Sen1 helicase

**DOI:** 10.1038/s41467-019-09560-9

**Published:** 2019-04-04

**Authors:** S. Wang, Z. Han, D. Libri, O. Porrua, T. R. Strick

**Affiliations:** 1 Molecular Motors and Machines group, Ecole normale supérieure, Institut de Biologie de l’Ecole normale supérieure (IBENS), CNRS, INSERM, PSL Research University, 75005 Paris, France; 2 Biomolecular Nanomanipulation group, Institut Jacques Monod, CNRS, University Paris Diderot, Sorbonne Paris Cité, F-75205 Paris, France; 3 Metabolism and Function of RNA in the Nucleus, Institut Jacques Monod, CNRS, University Paris Diderot, Sorbonne Paris Cité, F-75205 Paris, France; 4 Programme Equipe Labellisées, Ligue Contre le Cancer, 75013 Paris, France

## Abstract

Extrinsic transcription termination typically involves remodeling of RNA polymerase by an accessory helicase. In yeast this is accomplished by the Sen1 helicase homologous to human senataxin (SETX). To gain insight into these processes we develop a DNA scaffold construct compatible with magnetic-trapping assays and from which *S. cerevisiae* RNA polymerase II (Pol II), as well as *E. coli* RNA polymerase (*ec*RNAP), can efficiently initiate transcription without transcription factors, elongate, and undergo extrinsic termination. By stalling Pol II TECs on the construct we can monitor Sen1-induced termination in real-time, revealing the formation of an intermediate in which the Pol II transcription bubble appears half-rewound. This intermediate requires ~40 sec to form and lasts ~20 sec prior to final dissociation of the stalled Pol II. The experiments enabled by the scaffold construct permit detailed statistical and kinetic analysis of Pol II interactions with a range of cofactors in a multi-round, high-throughput fashion.

## Abstract (web summary)

Yeast’s Sen1 helicase is involved in the suppression of antisense transcription from bidirectional eukaryotic promoters. Here authors develop and utilize a quantitative single-molecule assay reporting on the kinetics of extrinsic eukaryotic transcription termination by the Sen1 helicase and a reaction intermediate in which the Pol II transcription bubble appears half-rewound.

## Introduction

Because eukaryotic promoters are intrinsically bidirectional^1^, molecular mechanisms have evolved to repress antisense transcription by promoting termination of the corresponding transcription elongation complex (TEC). The yeast Sen1 helicase, homologous to the human senataxin (SETX) helicase, is responsible for this activity, as well as for the regulation of synthesis and termination of a wide range of non-coding RNAs^2–4^. Over the course of the last few years the biochemical^5^ and structural^6^ properties of this enzyme have come into focus. At the same time, however, the detailed mechanism whereby Sen1 acts to remodel the TEC remains poorly understood, in part due to a lack of quantitative assays allowing one to measure and model the kinetics of TEC remodeling. Here, we develop and utilize a quantitative single-molecule assay reporting on the kinetics of extrinsic eukaryotic transcription termination by the Sen1 helicase.

Single-molecule assays have been used extensively to study transcription by both prokaryotic and eukaryotic RNA polymerases^7–11^. Although formation of prokaryotic TECs from a promoter requires only the classical *σ* cofactor, formation of eukaryotic TECs from a promoter requires a series of TFs and has typically been characterized by a low efficiency of successful initiation in vitro, raising obstacles to the mechanistic study of eukaryotic transcription at single-molecule resolution^11^. Promoter-dependent Pol II initiation has been sidestepped by pre-assembling Pol II on RNA/DNA scaffolds to form TECs with higher efficiency and study the mechanisms of elongation in high-resolution optical trapping systems^9,12^. However, because these are complex single-round assays in which each DNA molecule can be transcribed only once, relatively low data collection throughput represents a major limitation of such assays. This is particularly true for the study of interactions between extrinsic factors and transcribing Pol II.

Previously, we developed a single-molecule nanomanipulation assay^8,13^, in which DNA molecules were constrained at both ends in a magnetic trap, and using supercoiled DNA substrates we were able to study *Escherichia coli* RNAP transcription^8,14^ and transcription-related processes^15–17^ with ~bp spatial resolution and high data throughput. Here, we present a methodology based on a recyclable scaffold DNA construct^18,19^, which permits multi-round analysis of transcription, extending the spatial resolution and data throughput made possible by magnetic trap nanomanipulation to the eukaryotic transcription systems. We use this scaffold construct to characterize structure–function relations in the Pol II elongation complex and study important aspects of elongation, such as the action of the elongation factor TFIIS and the dynamics of R-loop formation. In addition, we employ our system to reveal important features of the transcription termination activity of the conserved helicase Sen1, a key actor in the control of pervasive transcription.

## Results

### Productive transcription from the scaffold

**Fig. 1:**
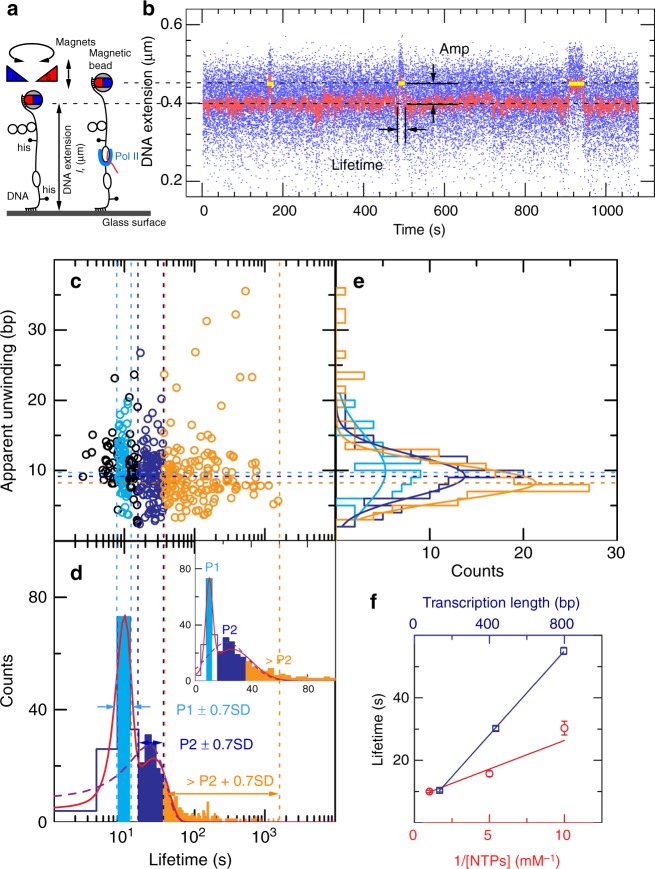
Experimental validation of Pol II initiation, elongation, and termination. **a** DNA containing an unpaired 12-base bubble flanked by *Ec his* terminators is tethered and torsionally constrained between a glass surface and a magnetic bead. **b** Time-trace obtained on negatively supercoiled Pol2-144-444-his construct shows the expected increases in DNA extension as small pulses, characterized by both their change in extension and their duration (Δ*l*, Δ*t*). Data were collected at 31.0802 Hz and all time traces were filtered at 0.5 Hz (red line). **c** 2D representation of transcription pulses reflecting their (Δ*l*, Δ*t*) coordinates (*N* = 407 events). **d** Lifetime projection of the 2D plot scaled in log is fit by a double-gaussian (red line, reduced chi-square is 0.75), yielding two peak durations: 10.0 ± 0.4 s (SEM) and 26.1 ± 1.6 s (SEM). Two sub-populations are selected based on the peak duration ± 0.7 SD: cyan, 10.0 ± 1.7 s (SD = 2.5 s for peak one); blue, 26.1 ± 10.1 s (SD = 14.5 s for peak two). Inset: linear scale of lifetime projection. The dashed violet line represents fitting to a single-gaussian with a reduced chi-square of 4.57. **e** Amplitude projection of the 2D plot sub-populations is fit to a single Gaussian (cyan line, giving a mean apparent unwinding of 9.9 ± 0.9 bp (SEM); blue line, 9.2 ± 0.3 bp (SEM); orange line; 8.3 ± 0.2 bp (SEM) in the blebbed bubble). **f** Dependence of the mean pulse lifetime on substrate length (blue) and inverse NTP concentration (red). Linear fitting of the length-dependence data (blue line) to the model *t* = *L*/*v* + *t*_0_, where *L* is the transcript unit length, *v* the velocity, and *t*_0_ the intercept gives *v* = 14.9 ± 0.4 bp/s and *t*_0_ = 1.3 ± 0.6 s. Nucleotide-dependence data are fit to a Michaelis–Menten model (red line) yielding affinity for NTPs *K*_M_ = 230 ± 66 μM and *V*_max_ = 16.7 ± 2.6 bp/s. Error bars represent SEM; typically at least 200 events were collected for each NTP concentration. Source data are provided as a Source Data file

The scaffold DNA we have developed is a 2.7 kb linear DNA containing a centrally located region of 12 bp in which the two DNA strands are not complementary and a permanent bubble is thus formed (Fig. 1a). Two *E. coli* *his* terminators, analogous to the *E. coli* *tR2* terminator previously shown to terminate Pol II transcription^20^, that are positioned flanking the permanent bubble, and oriented in such a way as to capture transcription initiated in either direction from the bubble. This construct thus contains both 144 and 444 bp transcription units, however the sequence of the bubble is such that Pol II can escape only in the direction of the shorter transcription unit when only ATP, UTP, and CTP are present, whereas it can escape bidirectionally when all four NTPs are present (Supplementary Fig. 1). For the purposes of single-molecule experimentation the construct is tethered between a magnetic bead and a glass surface, and extended and supercoiled using a magnetic trap. We can thus typically monitor ~50 DNA-tethered beads in a given field-of-view. Supercoiling the DNA under a low (*F* = 0.3 pN) extending force allows us to calibrate the topological properties of the scaffold, which are quite similar, if not identical, to those of a regular DNA molecule lacking the permanent bubble (Supplementary Fig. 2). At this force the torsional response of DNA consists in forming plectonemic supercoils for both positive and negative supercoiling, resulting in a typical decrease in DNA extension of about 50–55 nm/turn.

Addition of 1 nM Pol II, 1 μM 2-mer RNA (GpA), 1 mM NTPs, and 1 nM of the TFIIS antibacktracking factor to negatively supercoiled DNA led to discrete changes, or pulses, in DNA extension (Fig. 1b). These pulses reflect the action of individual Pol II molecules and consist in a sudden increase in DNA extension, followed by a constant-extension regime, followed by a sudden return (decrease) of DNA extension to its initial state. Each pulse was characterized by both its extension change, Δ*l*, and its duration, *T*, and a two-dimensional plot displaying each pulse as a pair of coordinates (Δ*l*, *T*) is presented Fig. 1c. The extension change is consistent with unwinding of approximately one turn of DNA, as expected for the Pol II transcription bubble, and is discussed in detail below. The distribution of pulse durations could be described by a double-Gaussian distribution with two peaks, the first at 10.0 ± 0.3 s (standard error of mean, SEM) and the second at 26.1 ± 1.6 s (SEM, Fig. 1d). The lifetime distribution was better fit by a double-Gaussian function (reduced chi-square is 0.75, Fig. 1d red line) rather than a single-Gaussian function (reduced chi-square is 4.57, see dashed violet line in Fig. 1d). The two peaks are consistent with Pol II transcribing in either direction from the bubble and terminating when it encounters a *his* terminator (144 and 444 bp away, respectively). A long tail which is not explained by the double-Gaussian fitting likely corresponds to Pol II molecules, which underwent pausing and backtracking during elongation (see below). The ratio in the two peak times (0.38 ± 0.03) is statistically consistent with the ratio of the lengths of the two transcripts produced from this construct (0.31). Taking into account the two different transcription unit lengths, we infer Pol II transcription rates from the permanent bubble at 13.4 ± 0.5 bp/s (SEM) and 16.6 ± 1.0 bp/s (SEM) for the short and long transcripts, respectively, consistent with expectations^9,12^. We infer that the rising edge of the pulses indeed correspond to blebbing of a transcription elongation bubble from the permanent bubble as depicted (Fig. 1a), and that the durations of the pulses essentially reflects the length of the transcripts generated by bidirectional transcriptional events initiated from the permanent bubble (see below). We note that the vast majority of pulses which initiate also terminate (*N*_Ter_ = 407), and a small fraction of readthrough events which initiated without termination showing an increase in DNA extension but never returning to baseline (*N*_nonTer_ = 62), allowing us to quantify the termination efficiency of Pol II on the strong *his* terminator from *E. coli* at 0.87 ± 0.04 (*N*_Ter_/(*N*_Ter_ + *N*_nonTer_)). This is comparable to the termination efficiency of *E. coli* RNAP on *his* terminator (0.78 ± 0.03, see ref. ^21^). When we replaced the *his* terminator with the *tR2* terminator (Supplementary Fig. 3F) we obtained a termination efficiency of 0.55 ± 0.05, in agreement with the termination efficiency observed in bulk for Pol II terminating on the *tR2* terminator^20^.

Based on this analysis of pulse duration we decomposed the 2D plot into three zones corresponding to synthesis of the short transcript (i.e. events lasting 10 ± 0.7 SD seconds), synthesis of the long transcript (i.e. events lasting 26 ± 0.7SD seconds), and synthesis most likely interrupted by pausing and backtracking (i.e. much longer than 26 s). The distribution of unwinding amplitudes for each zone is shown in the second projection of the 2D plot, Fig. 1e (cyan, blue, and orange curves, respectively). The extent of DNA unwinding in the transcription bubble during synthesis of short and long transcripts is essentially the same (9.9 ± 0.9 bp (SEM) and 9.2 ± 0.3 bp (SEM), respectively), and is significantly larger than that observed for the very long-lived transcription pulses (8.3 ± 0.2 bp (SEM)). These values are within a base-pair of those derived from structural analysis^22^. Overall these results suggest the very long-lived transcription pulses are associated with a deprecated transcription bubble, which may explain why they require so much more time to complete their task.

By also measuring pulse duration using constructs with only 444-base or 806-base transcription units and linearly fitting mean pulse duration vs. transcript unit length, we confirm that pulse lifetime reflects the length of the transcription unit (Fig. 1f) rather than time required for Pol II to completely blebb from the scaffold, or time required for Pol II to terminate transcription. By titrating NTPs from 1 mM to 100 μM, we observed a gradual decrease in the transcription rates of Pol II, which were well fitted with the Michaelis–Menten model giving *K*_m_ = 230 ± 66 μM and *V*_max_ = 16.7 ± 2.6 bp/s (Fig. 1f). These results are in quantitative agreement with prior single-molecule measurements of eukaryotic transcription elongation (see refs. ^9,12,23^ and Table 1).

**Table 1:** Comparison of Pol II transcription rates measured in this and other studies

	Pol II transcription rate
Single-molecule assay (this study)	16.7 ± 2.6 bp/s; saturated NTPs; *σ* = −0.027 (−sc) 14.9 ± 0.4 bp/s; [NTPs] = 1 mM; *σ* = −0.027 (−sc) 8.0 ± 0.3 bp/s; [NTPs] = 1 mM; *σ* = 0.016 (+sc)
Single-molecule assay by Galburt et al. ^9^	12.2 ± 4.5 bp/s; [NTPs] = 1 mM
Single-molecule assay by Palangat et al. ^12^	~25 nt/s with assisting force; [NTPs] = 1 mM ~23 nt/s with hindering force; [NTPs] = 1 mM
Bulk assay by Izban et al. ^31^	~1500 nt/min (+TFIIF); ~1300 nt/min (+TFIIF + TFIIX); ~1500 nt/min (+TFIIF + TFIIS); ~1300 nt/min (+TFIIF + TFIIX + TFIIS); [NTPs] = 1 mM
In vivo assay in human cells by Veloso et al. ^32^	~1.5 kb/min
	***E. coli*** **RNAP transcription rate**
Single-molecule assay (this study)	26.0 ± 2.6 bp/s (initiating from the bubble template and transcribing 144 bp); [NTPs] = 1 mM; *σ* = −0.027 (−sc) 35.9 ± 13.3 bp/s (initiating from the bubble template and transcribing 444 bp); [NTPs] = 1 mM; *σ* = −0.027 (−sc) 21.2 ± 0.7 bp/s (initiating from T5N25 promoter and transcribing 178 bp); [NTPs] = 1 mM; *σ* = −0.029 (−sc)
Single-molecule assay by Wang et al. ^7^	7.3 ± 3 bp/s averaged over assisting and hindering forces; [NTPs] = 0.2 mM
Single-molecule assay by Revyakin et al. ^8^	~17 bp/s; [NTPs] = 0.1 mM; *σ* = −0.021 (−sc) ~10 bp/s; [NTPs] = 0.1 mM; *σ* = 0.021 (+sc)

Furthermore, the experiments discussed so far all included 1 nM TFIIS, which serves to rescue backtracked Pol II^9,24^. In Supplementary Fig. 3 we show that the presence of TFIIS increases the likelihood that Pol II successfully completes transcription in a timely fashion, while its absence increases the fraction of Pol II molecules significantly delayed in their elongation task. Finally, Pol II transcription could also be observed using positively supercoiled DNA, although the initiation frequency was lower than that observed using negatively supercoiled DNA (Supplementary Fig. 4). We note that a topological change in the bubble associated with Pol II binding could be observed prior to escape from the bubble; independent of NTPs (data not shown), it presumably results from torsional rearrangement of the positively supercoiled bubble upon binding by Pol II. The pulse amplitude was essentially unchanged from that observed on negatively supercoiled DNA, however, the duration of pulses was longer, corresponding to a slower overall rate of bubble escape, transcription, and/or termination for positively supercoiled DNA (Supplementary Fig. 4F).

To show that the usefulness of this scaffold construct is not limited to eukaryotic transcription, we analyzed transcription of the Pol2-144-444-his construct but using *E. coli* core RNA polymerase (core *ec*RNAP, Supplementary Fig. 5). Transcription pulses were again observed and by applying the 2-D analysis described earlier we obtained an elongation rate on the order of 25 nt/s and a size for the transcription bubble on the order of 9 bases, consistent with prior measurements^8^.

We note that this assay also has use for the study of R-loop formation as demonstrated in Supplementary Fig. 6 and discussed in the Supplementary Text.

### Real-time analysis of transcription termination by Sen1

**Fig. 2:**
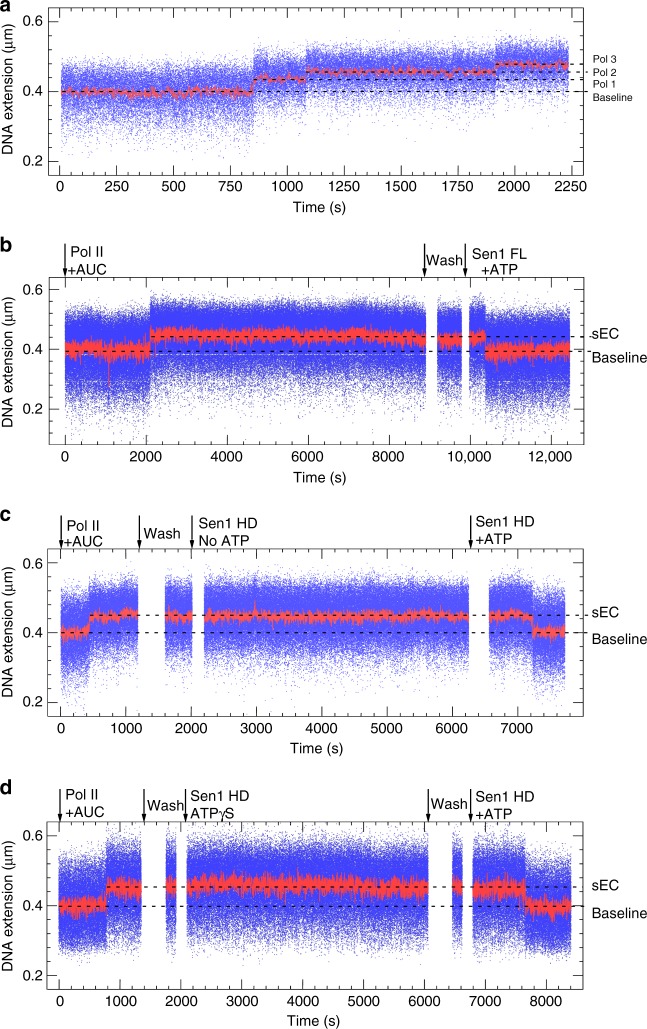
ATP-dependent removal of stalled Pol II by Sen1. **a** Time-trace showing successive extension increases as Pol II molecules accumulate on the DNA without terminating. **b** Time-trace showing a single Pol II can be removed by full-length Sen1 in the presence of ATP. Black arrows indicate moment of addition of indicated component. For the sake of clarity, DNA extension during additions is not shown. **c** Time-trace showing Pol II removal by Sen1 HD does not take place in the absence of ATP, but takes place in the presence of ATP. **d** Time-trace showing Sen1 HD does not remove stalled Pol II if provided with ATP-γ-S, a slowly hydrolyzable ATP analog

The Sen1 helicase from yeast plays an essential role in the control of pervasive termination by inducing non-coding transcription termination^2,3,5,25,26^. Previous studies have shown that Sen1 can translocate on the nascent RNA and induce dismantling of a stalled elongation complex in an ATP-dependent manner^5,26^. We took advantage of our system to further explore the mechanisms of action of Sen1. We generated stalled elongation complexes by using only ATP, UTP, and CTP, which allow Pol II to initiate and transcribe a 137-base G-less cassette but induce stalling at the first G of a G-stretch. As mentioned earlier the sequence of our DNA templates is designed so that the absence of GTP also restricted bidirectional transcription as this base is made limiting in the scaffold for the initiation of antisense transcription (see Supplementary Fig. 1). Under the magnetic trap, addition of 1 nM Pol II, 1 μM 2-mer RNA (GpA), 1 nM TFIIS, and 1 mM AUC caused the DNA extension to increase in a stepwise fashion as successive rounds of Pol II initiation and stalling on the construct result in a pile-up of polymerases (Fig. 2a). [A construct which stalled Pol II only 19 bp downstream of the bubble resulted in Pol II instability as the DNA extension frequently switched between the elongation state and the Pol II-binding state (Supplementary Fig. 7), suggesting Pol II backtracking into and emerging from the permanent bubble in a repetitive process.]

After washing out free components with buffer containing 1 mM AUC, the stalled Pol II is stable for many hours (Fig. 2a–d). Addition of 500 pM Sen1 and 1 mM ATP caused the DNA extension to return to its baseline value, indicating that Sen1 successfully displaced Pol II from the DNA (Fig. 2b)^3,5,25,26^. Although non-specific interactions between full-length Sen1 and DNA precluded exhaustive experimentation, we found that the helicase domain of Sen1 (Sen1 HD) recapitulated the remodeling activity of Sen1 (Fig. 2c), consistent with previous reports showing that the helicase domain is sufficient for termination in vitro^5,6^. This remodeling activity absolutely required both ATP binding and hydrolysis by Sen1 HD (Fig. 2d).

**Fig. 3:**
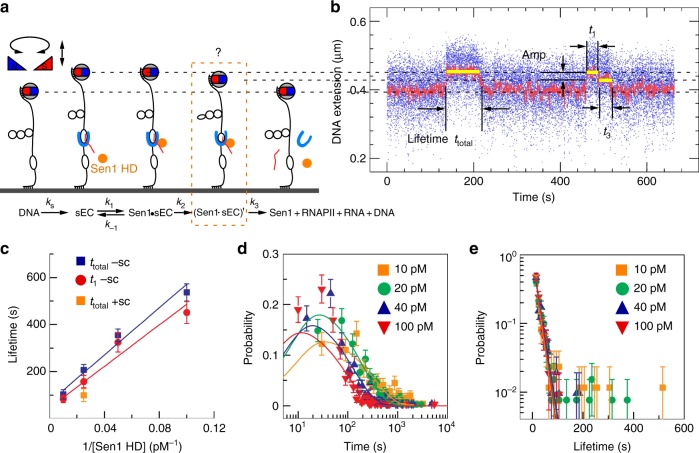
Titration of Sen1 HD activity. **a** Time-trace and accompanying sketch showing cycles of Pol II stalling and subsequent removal by Sen1 HD. The total lifetime of the transcription bubble is noted *t*_total_. **b** Typical pulses showing transcription initiation by Pol II and removal of resulting stalled Pol II by Sen1-HD. Sen1-HD, in a small majority of cases, removes stalled Pol II without any visible intermediates (e.g. first pulse, required time denoted *t*_total_). However, for a subset of ~40% of events, removal of Pol II from DNA can be seen to initiate after time *t*_1_ via formation of an intermediate in which the transcription bubble is apparently rewound by ~3.6 ± 0.1 bp (see Supplementary Fig. 8) (e.g. second pulse). Complete rewinding of the transcription bubble is observed after an additional time *t*_3_. **c** Mean lifetime of stalled Pol II on DNA as a function of Sen1-HD concentration under negative supercoiling (−sc, *σ* = −0.027) is well-fit to a Michaelis–Menten model (blue for *t*_total_ which is the total lifetime of all transcription bubbles; red for *t*_1_ which is the lifetime prior to intermediate formation when it can be detected). The orange data point represents the mean lifetime of stalled Pol II terminated by 40 pM Sen1 HD for positive supercoiling (+sc, *σ* = 0.016, *n* = 37 events). Error bars represent SEM. **d** Distribution of lifetimes (*t*_total_) of Pol II on DNA as a function of Sen1-HD concentration is well-fit to a single-molecule recasting of the Michaelis–Menten equation (see Supplementary notes) giving a chi-square of 211.5 and a reduced chi-square of 1.47. **e** Distribution of duration *t*_3_ is well-fit to single-exponential statistics across the concentration range tested, giving time constants of 16.4 ± 2.4 s (SEM, *n* = 86), 18.1 ± 1.8 s (SEM, *n* = 131), 19.0 ± 2.4 s (SEM, *n* = 105), and 16.3 ± 1.9 s (SEM, *n* = 107) for 10, 20, 40, and 100 pM, respectively, of Sen1 HD. Source data are provided as a Source Data file

We next titrate Sen1 HD concentrations against Pol II by incubating 1 nM Pol II, 1 μM 2-mer RNA (GpA), 1 nM TFIIS, 1 mM AUC, 1 mM ATP, and Sen1 HD together. Sen1 HD concentration was progressively increased from 10 to 100 pM and we observed a gradual reduction in the mean lifetime of stalled Pol II (Fig. 3a–c). These data could be fit to a simple Michaelis–Menten model, giving *K*_m_ = 70 ± 25 pM and *k*_CAT_ = 0.0142 ± 0.0051 s^−1^. The latter parameter must be corrected for the *t*_stall_ ~ 9 s that Pol II spends transcribing the 137-base G-less cassette before it stalls (stalling rate *k*_s_ = 1/*t*_stall_), and so we ultimately obtain as reaction parameters *K*_m_ = 83 ± 36 pM and *k*_CAT_ = 0.0165 ± 0.0069 s^−1^ (i.e. 1/*k*_CAT_ ~ 60 s). This correction of *t*_stall_ is required because transcribing Pol II does not appear to be a target for Sen1 action^3,26^. We obtained similar results when we applied the single-molecule casting of Michaelis–Menten to the mean lifetime of stalled Pol II and performed global fitting on all observed events obtained at different Sen1 HD concentrations (Fig. 3d)^27^. For these fits we constrained *k*_CAT_ to the value obtained from the Michaelis–Menten model and obtained *k*_1_ = 2.18 ± 0.05 × 10^9^ M^−1^ s^−1^ and *k*_−1_ = 0.0719 ± 0.007 s^−1^ (see kinetic scheme below). Combined with *k*_CAT_ this gives *K*_m_ = 41 ± 3 pM, consistent with estimates of *K*_m_ from the classical Michaelis–Menten model.

Detailed inspection of the time-traces showed that a significant fraction (on the order of 40%, see Table 3) of displacement events showed extra detail in the remodeling time trace with the formation of a DNA state with intermediate extension prior to final displacement of Pol II (see for instance the second event in Fig. 3b). This intermediate state was never observed to return to the state corresponding to stalled Pol II. This indicates that Pol II undergoes an irreversible rearrangement prior to full release from DNA. We therefore rewrite the overall reaction as consisting in rapid binding/unbinding of Sen1 to the stalled elongation complex, sEC, so as to form an enzyme–substrate complex, Sen1·sEC, which is converted via an initial catalytic step into an intermediate (Sen1·sEC)’, before being dissociated from DNA in a final catalytic step:

1 $${\mathrm{Sen1}} + {\mathrm{sEC}}\ \mathop{\leftrightarrows}\limits_{{{k}_{ - 1}}}^{{{k}_1}}\ {\mathrm{Sen1}}	 \cdot {\mathrm{sEC}}\mathop{\longrightarrow}\limits^{{{k}_2}}\left( {{\mathrm{Sen1}} \cdot {\mathrm{sEC}}} \right)\prime \mathop{\longrightarrow}\limits^{{{k}_3}}{\mathrm{Sen1}} \\ 	 \hskip -18pt + {\mathrm{RNAP}} + {\mathrm{DNA}} + {\mathrm{RNA}}$$

The lifetime distribution for this intermediate state (Sen1·sEC)’ obeyed single-exponential statistics and did not vary significantly with Sen1 HD concentration (Fig. 3e). Averaging over the four concentrations assayed we obtain a mean of 1/*k*_3_ = 17.5 ± 1.3 s (SD, see legend to Fig. 3e), i.e. *k*_3_ = 0.0572 ± 0.0043 s^−1^.

We then used the classical Michaelis–Menten model to analyze the concentration-dependence of the initial time during which stalled Pol II waits before forming this intermediate state (Fig. 3b). Modeling those events which display the intermediate gives *K*_m_ = 96 ± 45 pM and *k*′_CAT_ = 0.0219 ± 0.0097 s^−1^, where 1/*k*′_CAT_ = 1/*k*_2_ + 1/*k*_s_. We thus obtain an estimate for *k*_2_ = 0.0277 ± 0.0156 s^−1^ (or 1/*k*_2_ = 40 ± 20 s). We note that for *k*_CAT_ derived from Michaelis–Menten analysis of the total time 1/*k*_CAT_ = 1/*k*_S_ + 1/*k*_2_ + 1/*k*_3_, and that the value we obtain here for *k*_2_ is consistent with that obtained in the prior analysis of the total time. In other words, the rate-limiting kinetic step obtained by classical Michaelis–Menten analysis of the total duration of events (60 s) is roughly equal to the sum of 1/*k*_2_, the rate-limiting kinetic step obtained by classical Michaelis–Menten analysis of formation of (Sen1·sEC)’ (40 s), and 1/*k*_3_, the rate at which (Sen1·sEC)’ is directly observed to undergo resolution (20 s) (see Table 2 for an overview of fitting parameters).

**Table 2:** Summary of fitting parameters

	*K*_m_	*k*_CAT_		
*t*_total_ MM	70 ± 25 pM	0.0142 ± 0.0051 s^−1^		
	*K*_m_	*k*_CAT_	1/*k*_s_	
*t*_total_ MM corrected for *k*_s_	83 ± 36 pM	0.0165 ± 0.0069 s^−1^	9.65 ± 0.34 s	
	*K*_m_	*k*_CAT_	*k*_1_	*k*_−1_
*t*_total_ single-molecule MM	41 ± 3 pM	constrained as 0.0142 s^-1^	2.18 ± 0.05 × 10^9^ M^−1^ s^−1^	0.0725 ± 0.007 s^−1^
	*K*_m_	*k*’_CAT_	*k*_2_	*k*_3_
*t*_1_ MM	96 ± 45 pM	0.0219 ± 0.0097 s^−1^	0.0277 ± 0.0156 s^−1^	0.0572 ± 0.0043 s^−1^

This suggests that the (Sen1·sEC)’ intermediate is always formed, even if it is not directly detected. However, we only observe the intermediate in 1/3 of events (see Table 3). The amplitude and lifetime of the intermediate imply that we are missing another 1/3 of events given the measurement resolution (Gaussian noise on the bead is ~20 nm at 31 Hz and the bead cutoff frequency is ~4 Hz). It is thus possible that a minority fraction of complexes dissociates directly without formation of an intermediate.

**Table 3:** Summary of the number of events displaying or not displaying intermediates

[Sen1 HD] (pM)	Events displaying intermediate	Predicted number of events lacking intermediate	Actual number of events lacking intermediate	Total number of events
10	86 (35.1%)	60 ± 16	159	245
20	131 (43.2%)	90 ± 17	172	303
40	105 (37.0%)	68 ± 15	179	284
100	107 (36.3%)	89 ± 19	188	295

The intermediate could be identified because it corresponds to an extension state distinct from the elongation state and consistent with partial rewinding of the transcription bubble. Thus, for negatively supercoiled DNA, the extent of DNA rewinding which takes place upon formation of the intermediate state is 3.6 ± 0.1 bp (Supplementary Fig. 8D). To determine whether or not this corresponds to true DNA unwinding rather than DNA bending/compaction in the intermediate, we carried out experiments using Pol II stalled on positively supercoiled DNA at 40 pM Sen1 HD (Supplementary Fig. 8A–C). In these conditions Pol II was removed from positively supercoiled DNA nearly twice as fast as from negatively supercoiled DNA (see Fig. 3c, orange point). In addition, no intermediate could be detected here, possibly as a result of shorter dwell times on positively supercoiled DNA. If the intermediate had consisted in a bent/wrapped DNA state, then in principle this would not be affected by supercoiling and should appear as a long-lived (~20 s) decrease in DNA extension for positively supercoiled DNA substrate. As no such state is detected, we conclude that the intermediate Pol II state observed during remodeling by Sen1 corresponds either to a partially rewound transcription bubble, or to a fully rewound transcription bubble (and displaced Pol II) but with Sen1 remaining on the DNA and distorting it.

Prior results obtained from bulk assays have shown that the activity of Sen1 is essentially dependent on the presence of the RNA in the stalled elongation complex^26^. To verify this in the single-molecule assay we repeated our measurements in the presence of Rnase A (0, 50, 100 μg/ml). The fraction of Pol II molecules successfully terminated by Sen1 HD in these conditions decreased from 95% to 30% in the presence of RnaseA (Supplementary Fig. 8E), in quantitative agreement with ref. ^26^.

**Fig. 4:**
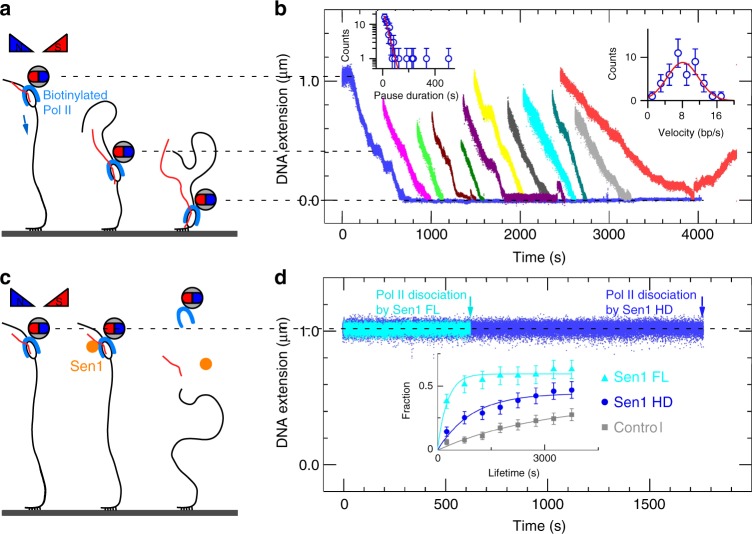
Stalled Pol II is fully dissociated from DNA upon action of Sen1 FL or Sen1 HD. **a** Schematic of the assay. Biotinylated Pol II is stalled on DNA, tethered to a magnetic bead, and placed in the magnetic trap. Transcription against a 1 pN force is restarted by addition of TFIIS and NTPs (24 out of 58 molecules resumed transcription). **b** Time-trace showing processive transcription by Pol II with interruptions due to long-lived pauses. Pauses shorter than ~10 s cannot be detected despite data filtering on the 2 s timescale. Detectable pauses are distributed according to single-exponential statistics with a mean of 29 ± 5 s (SEM, *n* = 67; left inset). When pauses longer than 10 s are removed, the velocity distribution can be fit to a Gaussian (red line) with mean velocity of 8.1 ± 0.6 bp/s (SEM, *n* = 43; right inset). **c** Sketch of the assay in the presence of Sen1 FL or Sen1 HD. **d** Time-trace showing release of stalled Pol II by 500 pM Sen1 FL (cyan; 76 Pol II molecules released out of 121), 500 pM Sen1 HD (blue; 52 Pol II molecules released out of 111). In controls carried out without Sen1 only 35 of 127 Pol II molecules released spontaneously. Inset shows the distribution of time elapsed between addition of translocase and release of Pol II. By fitting to a cumulative expression of single exponential function: *f*(*t*) = A*(1–exp(−*t* /*t*_0_)), where *A* is the fraction of molecules which ultimately dissociate, *t*_0_ is the time constant of this dissociation, we obtained the mean time for releasing stalled Pol II as 2935 ± 1840 s (SEM, *n* = 35) for no Sen1, 265 ± 69 s (SEM, *n* = 77) for Sen1 FL, and 963 ± 278 s (SEM, *n* = 52) for Sen1 HD. The final fraction of dissociation is 0.37 ± 0.15 for no Sen1, 0.59 ± 0.03 for Sen1 FL, and 0.45 ± 0.05 for Sen1 HD. Source data are provided as a Source Data file

Finally, to determine whether or not stalled Pol II is fully released from DNA upon remodeling by Sen1, we implemented a translocation assay^16,17^ in which the Pol II-DNA system is assembled in the magnetic trap by tethering one end of the DNA to the glass surface and attaching the magnetic bead directly to the Pol II (via a biotin moiety introduced into Pol II) (Fig. 4a). Since a transcribing Pol II molecule can be stalled and backtracked by a ~8 pN hindering force^9^, a low extending force (1 pN) was applied to avoid force-induced effects. A Pol II molecule was stalled at +137 in the presence of AUC and restarted transcription towards the glass surface by addition of NTPs (Pol2-G-less-137-T construct, which sustains transcription towards the surface only). Processive Pol II elongation can directly be observed in this assay as the enzyme carries the bead with it towards the surface (Fig. 4b). Different transcription run lengths were observed because some Pol II molecules restart during NTP addition. Pol II stalling can also be accomplished on this substrate (Fig. 4c), and upon addition of either Sen1 FL or Sen1 HD we observe Pol II dissociation as an irreversible and instantaneous loss of the magnetic bead (Fig. 4d).

## Discussion

The development of a scaffold transcription construct based on the DNA supercoiling assay provides a way of measuring eukaryotic termination-related processes independently of TFs and in a multi-round fashion allowing for observation of a succession of individual Pol II molecules. This approach is compatible with both eukaryotic and prokaryotic transcription, however, it is not compatible with promoter-dependent studies. Additional work will be required to ascertain whether this approach is compatible with transcription-factor-dependent studies. Here the complex is viewed through the formation and dissolution of the topological transcription bubble universally formed by RNA polymerases. We thus characterize the mechanism and kinetics of dissociation of a stalled elongation complex by the Sen1 helicase responsible for termination of non-coding transcription. Our findings include the observation that Sen1 HD is remarkably affine for the stalled elongation complex and functions very efficiently even at sub-nanomolar concentrations (*K*_M_ ~ 50–100 pM). Nevertheless, the helicase is three times likelier to dissociate spontaneously from stalled Pol II than it is to succeed in displacing Pol II. From a catalytic perspective, Pol II remodeling by Sen1 helicase is slow and involves the formation of a succession of complexes, including an intermediate Sen1–Pol II complex in which the transcription bubble is apparently half-rewound. Sen1 requires ATP binding and hydrolysis to generate this intermediate. The intermediate forms slowly, appearing a full ~40 s after binding of Sen1 to Pol II, and has a relatively long lifetime of ~20 s before the Pol II dissociates from the DNA. The overall process is thus characterized by slow catalysis on the minute timescale. Taken together these results suggest that the target for Sen1 in vivo is a Pol II molecule which has come to be immobilized. The combination of this method with single-molecule fluorescence as in the NanoCOSM assay (for nanomanipulation and colocalization of single-molecules^16,17^) will allow for the direct observation of fluorescently labeled effectors of eukaryotic transcription binding and unbinding from Pol II as it works to transcribe DNA.

## Methods

### Plasmids and sequences

A 2-kb DNA fragment was PCR amplified from the *Thermus aquaticus RPOC* gene, with primers RPOC_F and RPOC_R (see primer list in Supplementary Table) containing, respectively, an XbaI and an SbfI site separately. This fragment contains a unique, centrally located, and unique KpnI site for insertion, in later cloning steps, of specific transcription units. This 2-kb fragment was subcloned through the XbaI and SbfI sites into the pUC18 polylinker to build the backbone plasmid. The unique EcoRI of pUC18 was destroyed by first digesting with EcoRI, then blunting the overhang with the Quick Blunting Kit (NEB), and finally ligating the blunt ends together with the Quick Ligation Kit (NEB) prior to cloning.

We designed and obtained via gene synthesis (Eurofins Genomics) the DNA sequence Pol2-144-444-his (see Supplementary Notes) and which is flanked by KpnI sites (red). This sequence presents a region delimited by unique HindIII (blue) and SpeI (green) sites and into which one can insert the permanently mispaired bubble used as a scaffold for Pol II initiation. This modular region is flanked on either side by the *his* transcription termination sequence of *E. coli* (underlined). The sequences to be transcribed and which are contained between the HindIII and *his* terminator on one side, and the SpeI and *his* terminator on the other side, are from the *E. coli* lacZ gene and are 144 and 444 bp long, respectively. The Pol2-144-444-his fragment was cloned into the backbone plasmid via its KpnI sites. The resulting DNA plasmid, named pUC18 Pol2-144-444-his, was purified from an overnight culture of *E. coli* using the Nucleobond Xtra Midi Plus kit for alkaline lysis and ion exchange (Macherey-Nagel).

We designed and obtained via gene synthesis (Eurofins Genomics) the DNA sequence Pol2-444-444-his (see Supplementary notes). This sequence presents a region delimited by unique HindIII (blue) and SpeI (green) sites, and into which one can insert the permanently mispaired bubble used as a scaffold for Pol2 initiation. It differs from the Pol2-144-444-his sequence described above in that both sequences to be transcribed and which are contained between the HindIII site and *his* terminator on one side, and the SpeI site and *his* terminator on the other side, are 444 bp long. Unique AvrII (bold, light green) and MluI (bold, purple) restriction site are also present in this sequence for added modularity. This fragment was cloned into the backbone plasmid via its KpnI sites. The resulting DNA plasmid, named pUC18 Pol2-444-444-his, was purified from an overnight culture of *E. coli* using the Nucleobond Xtra Midi Plus kit for alkaline lysis and ion exchange (Macherey-Nagel).

We constructed the DNA sequence Pol2-806-806-his via the following procedure. We first PCR amplified two 362 bp fragments from Pol2-444-444-his plasmid using the primers 444_LF and 444_LR (containing, respectively, unique AvrII and SpeI sites shown underlined) and 444_RF and 444_RR (containing, respectively, unique AscI and MluI sites shown underlined).

The first PCR fragment was digested with AvrII and SpeI and cloned into the pUC18 Pol2-444-444-his plasmid via through its AvrII site. The desired orientation of the 362 bp fragment was selected by sequencing and maintained the AvrII site at the 5′ end of the sequence. Then, the second PCR fragment was digested with AscI and MluI and cloned into the pUC18 Pol2-444-444-his plasmid through its MluI site. The desired orientation of the 362 bp fragment was selected by sequencing and maintained the MluI site at the 3′ end of the sequence. The resulting plasmid, named pUC18 Pol2-806-806-his, was expressed and purified as above.

The Pol2-G-less-cassette sequence specified below was designed and obtained by gene synthesis (see Supplementary notes). It contains two KpnI sites (red) and a region flanked by HindIII (blue) and EcoRI (yellow) sites. The sequence also contains unique SpeI (green) and MluI (bold, purple) sites. Downstream of the EcoRI site is a G-less sequence. This sequence was cloned into the backbone plasmid through its KpnI sites. The resulting plasmid, named pUC18 Pol2-G-less cassette, was expressed and purified as above.

We designed and obtained via gene synthesis (Eurofins Genomics) the DNA sequence T5N25-178his (see Supplementary notes). This sequence presents a T5N25 promoter sequence (blue) followed by 178 bp transcription unit and a *his* terminator. This fragment was cloned into the backbone plasmid via its KpnI sites. The resulting DNA plasmid, named pUC18 T5N25-178his, was purified from an overnight culture of *E. coli* using the Nucleobond Xtra Midi Plus kit for alkaline lysis and ion exchange (Macherey-Nagel).

### DNA constructs for tethered-DNA supercoiling assays

We prepared three constructs containing a scaffold bubble flanked by a *his* terminator on each side. The Pol2-144-444-his construct sustains bidirectional transcription from the bubble; in one direction an *E. coli his* terminator lies 144 bp away; in the other direction a *his* terminator lies 444 bp away. The Pol2-444-444-his construct differs in that the *his* terminators are each located 444 bp from the bubble, while for the Pol2-806-806-his construct the *his* terminators are each located 806 bp from the bubble.

First, plasmids pUC18 Pol2-144-444-his, pUC18 Pol2-444-444-his, and pUC18 Pol2-806-806-his were digested 3 h with HindIII and SpeI restriction enzymes (New England Biolabs). The ~20 bp fragment contained between the HindIII and SpeI sites was removed by agarose gel electrophoresis and the longer (~5 kb) fragment extracted from the gel (Macherey-Nagel PCR and Gel Extraction Kit).

5′ phosphorylated oligonucleotides non-tem1 and tem1 (Eurofins Genomics) were annealed to form a scaffold dsDNA oligo by combining to 50 μM each in 1x PBS, heating to 95 ^o^C for 2 min, and then cooling to room temperature over a 2 h period. The regions underlined in the oligos form an unpaired region after annealing the scaffold, and the ends of the annealed scaffold oligo are compatible with ligation into HindIII and SpeI overhangs.

The Pol2-144-444-his, Pol2-444-444-his, and Pol2-806-806-his constructs for tethered-DNA supercoiling assays were prepared by overnight ligation at room temperature of the scaffold oligo into the pre-digested and purified pUC18 Pol2-144-444-his, Pol2-444-444-his, or Pol2-806-806-his plasmids. The ligation product was then purified with a NucleoSpin Gel and PCR Clean-Up kit (Macherey-Nagel) and then digested with XbaI, SbfI, and AseI (New England Biolabs). The desired DNA fragment (2.7 kb for the Pol2-144-444-his construct, 3 kb for the Pol2-444-444-his construct, and 3.4 kb for Pol2-806-806-his construct) was isolated by gel purification and extraction. The DNA molecules were then ligated to 1 kb DNA fragments modified with multiple biotin groups through the XbaI site and to 1 kb DNA fragments modified with multiple digoxigenin groups through the SbfI site. The modified DNA fragments were synthesized via PCR amplification in the presence of dUTP-biotin and dUTP-digoxigenin, respectively (Strick 2005 nmeths).

The T5N25-178his construct was prepared by digesting the T5N25-178his plasmid with XbaI, SbfI, and AseI (New England Biolabs) and isolating via gel purification and extraction. The produced 2.2 kb DNA were then ligated to 1 kb DNA fragments modified with multiple biotin groups through the XbaI site and to 1 kb DNA fragments modified with multiple digoxigenin groups through the SbfI site.

### Stalling constructs for tethered-DNA supercoiling assays

Two constructs containing a scaffold bubble and a G-less transcription cassette were prepared. The Pol2-G-less-137 construct sustains unidirectional transcription from the scaffold bubble and stalls Pol2 137 bp from the bubble. The Pol2-G-less-19 construct sustains unidirectional transcription from the scaffold bubble and stalls Pol2 19 bp from the bubble.

First, plasmid pUC18 Pol2-G-less cassette was digested 3 h with HindIII and EcoRI restriction enzymes (New England Biolabs). The ~20 bp fragment contained between the HindIII and EcoRI sites was removed by agarose gel electrophoresis and the longer (~5 kb) fragment extracted from the gel (Macherey-Nagel PCR and Gel Extraction Kit).

5′ phosphorylated oligonucleotides non-tem1 and tem2 (Eurofins Genomics) were annealed to form a second scaffold dsDNA oligo as above. The regions underlined in the oligos form an unpaired region after annealing the scaffold, and the ends of the annealed scaffold oligo are compatible with ligation into HindIII and EcoRI overhangs.

The two stalling constructs (Pol2-G-less-137 and Pol2-G-less-19) for tethered-DNA supercoiling assays were prepared in a similar fashion. For the Pol2-G-less-137 construct, the second scaffold oligo (non-tem1 and tem2) was ligated into the pre-digested Pol2-G-less-cassette plasmid prepared as above. To assemble the Pol2-G-less-19 construct, the first scaffold oligo (non-tem1 and tem1) was ligated into Pol2-G-less-cassette plasmid pre-digested as above but with the HindIII and SpeI restriction enzymes. Remaining steps of the assembly procedure were performed as described above.

### Stalling constructs for tethered-Pol II translocation assays

The Pol2-G-less-137-T stalling construct for tethered-Pol II translocation assays was prepared following the same procedures as Pol2-G-less-137 construct preparation, up to and including overnight ligation of the scaffold bubble into the plasmid and purification of the ligation reaction from enzymes using a Macherey-Nagel PCR and Gel Extraction Kit. The purified ligation product was then digested with XbaI and NcoI (the NcoI site is located between the XbaI site and the inserted bubble scaffold) and the target DNA fragments (~4.6 kb) was isolated by gel purification. The target DNA molecules were then ligated to 1 kb DNA fragments modified by multiple digoxigenins via the XbaI site and in the presence of NcoI-restriction enzyme. When the resulting DNA is tethered to an antidigoxigenin-treated glass surface via the digoxigenin groups, transcription from the scaffold directs Pol II towards the glass surface.

2-mer RNA (5′-GpA) was purchased from TriLink Bio Technologies and 9-mer RNA (5′-ACACGGCGA) was from Dharmacon/GE Healthcare.

Surfaces used for single-molecule experiments were prepared and derivatized with anti-digoxigenin^28^.

### Tethered-DNA supercoiling assays

DNA molecules were first attached to 1-μm-diameter streptavidin-coated superparamagnetic beads (Dynabeads MyOne Streptavidin C1; Life Technologies) and then to a glass surface functionalized with anti-digoxigenin. The glass surface was placed atop a homemade magnetic trap which monitors and analyzes the position of the tethered superparamagnetic bead with the PicoJai software package (PicoTwist SARL). Data were collected at video rate (31.0802 Hz) and filtered at 0.5 Hz. The standard deviation for bead fluctuations at 31 Hz was *s* = 20 nm and the bead cutoff frequency was 4 Hz. Data were processed using custom routines in the Xvin software subsuite of PicoJai. Experiments were performed in standard transcription buffer containing 20 mM K-HEPES pH 7.5, 150 mM K-Glut, 8 mM Mg(Ac)_2_, 0.5 mg/ml BSA, 0.1% w/v Tween 20, 2 mM DTT, and 10 μM ZnCl_2_ at 28 °C. DNA molecules were extended and torsionally constrained (*F* = 0.3 pN, where 1 pN = 10^−12^ N; superhelical density = −0.027 or −7 turns for negative supercoiling; 0.016 or +4 turns for positive supercoiling).

Continuous-tracking and pulse-chase methodologies were used for tethered-DNA supercoiling assays, showing identical results in terms of quantitative analysis and thus simply representing different levels of optimization of these measurements^8,15^.

Continuous tracking methodology was used to test Pol II transcription initiating from the bubble, in which Pol2-144-444-his construct was used for testing Pol II initiates and transcribes in either direction from the bubble by addition of 1 nM Pol II, 1 μM 2-mer RNA, 1 mM NTPs containing 1 mM each of ATP, UTP, GTP, and CTP, and 1 nM TFIIS. Pol2-144-444-his construct, Pol2-444-444-his construct and Pol2-806-806-his construct were used for testing transcription length effects on Pol II transcription, in which 25 nM TFIIS was used. NTPs concentrations (0.1, 0.2, and 1 mM) were titrated by using Pol2-144-444-his construct. For testing TFIIS activities, the Pol2-444-444-his construct was used with 0 or 25 nM TFIIS. For Sen1 termination experiments, Pol2-G-less-137 stalling construct, 1 nM Pol II, 1 μM 2-mer RNA, 2 mM ATP, and 1 mM each of UTP and CTP, 1 nM TFIIS, and various Sen1 HD concentrations (10, 20, 40, and 100 pM) were used.

The pulse-chase methodology has been described previously^17^. Single-round ‘pulse-chase’ assays are optimal for measuring the interactions of partner proteins with a single RNA polymerase stalled on DNA, without interference or added measurement noise generated by free RNA polymerase in solution. To stall Pol2 on nanomanipulated, supercoiled DNA, we first added 1 nM Pol II, 1 μM 2-mer RNA, 1 mM AUC, and 1 nM TFIIS and incubated for 2000 s. Longer incubation time was used to stall multiple Pol II molecules on the same DNA construct. We then washed away free components with transcription buffer, and then added 500 pM Sen1 FL or 40 pM Sen1 HD, along with 1 mM ATP, to measure the displacement of stalled Pol2. For measurements assaying the role of ATP binding and hydrolysis in Sen1 activity, 40 pM Sen1 HD was added to stalled Pol2 either in the absence of ATP or in the presence of 1 mM ATPγS. Positive controls of negative controls were conducted on the same molecules by final addition of 40 pM Sen1 HD and 1 mM ATP.

For the RNaseA assays, we assembled reactions on the Pol2-G-less-cassette construct by mixing 1 nM Pol II + AUC (1 mM each), 40 pM Sen1 HD + ATP (1 mM), and RNase A (0, 50, or 100 μg/ml, Thermo Fischer).

### Tethered-Pol II translocation assays

Pol II was stalled on the Pol2-G-less-137-T construct at +137 (the first hybridized base pair downstream of the bubble was counted as +1) by incubating 8 pM DNA with 1 nM biotin-labeled Pol II, 1 μM 2-mer RNA, 1 mM AUC containing 1 mM each of ATP, UTP, and CTP, and 1 nM TFIIS at 28 °C for 30 min; followed by mixing with streptavidin-coated beads and deposition onto the glass surface. After washing away free components, a 1 pN force was applied to gently extend the DNA molecules. Pol2 transcription was restarted by adding the four nucleotides (1 mM each) alone or with 1 nM TFIIS. For Sen1 termination experiments 500 pM Sen1 HD or Sen1 FL were added along with 1 mM ATP.

Time-traces were analyzed using the PicoJai software suite (PicoTwist SARL) and transcription pulses were manually assigned and analyzed for duration and amplitude.

### Bulk assays

DNA oligos (non-tem3 and tem3, Eurofins Genomics, FPLC purified, see Supplementary Table) were mixed in equimolar amounts (10 μM each) in 10 mM Tris pH 7.5, 50 mM NaCl buffer, heated to 95 °C for 2 min and cooled slowly to room temperature to form the transcription template containing a permanent unpaired region (or bubble, highlighted in yellow). A final concentration of 0.1 mg/ml BSA was added after annealing the duplex.

Final concentrations of 5 nM annealed duplex, 50 nM Pol2, 10 μM UTP, 0.33 μM α-radiolabeled UTP, 1 mM ATP + CTP or 1 mM ATP + GTP + CTP, and 1 μM GpA were incubated in transcription buffer (20 mM Tris–HCl pH 7.5, 100 mM NaCl, 8 mM MgCl_2_, 10 μM ZnCl_2_, 10% (v/v) glycerol, 2 mM DTT) at 28 °C for 20 or 60 min for Pol2 transcription. After the reaction, the radiolabeled transcript was migrated on denaturing polyacrylamide sequencing gels.

### Protein purification

*S. cerevisiae* RNA Polymerase II (Pol II) was purified from a strain that expresses a His_6_-tagged version of Rpb3p essentially as previously described^6^. Briefly, the cell pellet was resuspended in lysis buffer (20 mM Tris–HCl pH 8, 150 mM KCl, 10% (v/v) glycerol, 10 mM ZnCl_2_, 10 mM DTT) and lysed using a Carver press. After clarification, the protein extract was precipitated with 40% ammonium sulfate, and subjected to Ni-affinity chromatography (Ni-NTA, Qiagen) and then anion exchange chromatography (Mono-Q 5/50 GL, GE Healthcare). The fractions of interest were dialyzed against Pol2 storage buffer (10 mM HEPES pH 7.9, 40 mM (NH_4_)_2_SO_4_, 10 mM ZnCl_2_, 10% (v/v) glycerol, 10 mM DTT) and stored at −80 °C.

Biotinylated RNA Pol II was prepared by incubating 50 mg of Pol II bearing the AviTag and 10 µg of BirA biotin ligase protein in 200 µl reaction buffer (10 mM HEPES pH 7.9, 40 mM (NH_4_)_2_ SO_4_, 5 µM ZnCl_2_ 2.5 mM DTT, 5% (v/v) glycerol, 10 mM MgAc-ATP, and 0.1 mM biotin) for 5 h at 4 °C, followed by dialysis against Pol2 storage buffer and stored at −80 °C.

*E. coli* RNA polymerase (*ec*RNAP) was purified as previously described^16^. Briefly, the cell pellet was resuspended in lysis buffer (20 mM Tris–HCl, pH 8.0, 500 mM NaCl, 5% glycerol) and lysed using Emulsiflex C5, Avestin. After clarification, the protein was loaded onto 10 ml of nickel-chelated metal-affinity resin (HiTrap Chelating, GE Healthcare) and then subjected to 10 ml of heparin resin (HiTrap Heparin, GE Healthcare). The fractions containing core RNAP were pooled and half the pooled volume was saturated with recombinant σ^70^ (prepared as in ref. ^29^) and dialyzed overnight into dialysis buffer (20 mM Tris–HCl, pH 8.0, 200 mM NaCl, 0.1 mM EDTA, 1 mM DTT, and 50% glycerol) before flash freezing and storage at −80 °C (to make holoenzyme *ec*RNAP). The other half-volume was not saturated with σ^70^ (i.e. core *ec*RNAP) but directly aliquoted, flash frozen, and stored at −80 °C.

Sen1 FL was purified from yeast strain DLY1774 (derived from W303) as previously described^5^, which overexpresses N-terminal TAP-tagged Sen1 FL from the *GAL1* promoter. Cell pellet from 4 l of YPA culture containing 2 g/l of galactose at OD_600_ ≈ 2 was resuspended in AGK buffer (10 mM HEPES pH 7.9, 1.5 mM MgCl_2_, 200 mM KCl, 10% (v/v) glycerol, 0.5 mM DTT) containing protease inhibitors (2 mM AEBSF, 2 mM benzamidine, and EDTA-free Protean from Roche) and lysed using a Retsch MM301 Ball Mill. The suspension was clarified by centrifugation (30 min at 30,000×*g* at 4 °C) and treated with RNaseA + T1 (10 mg/ml) for 20 min at 25 °C before loading onto IgG-sepharose beads (GE Healthcare) pre-equilibrated with IPP150 buffer (10 mM Tris–HCl pH 7.5, 150 mM NaCl, 0.1% NP40, 5% (v/v) glycerol). Beads were profusely washed with IPP150 and then with IPP500 (as IPP150 but containing 500 mM NaCl) and then with TEV cleavage buffer (10 mM Tris–HCl pH 7.5, 150 mM NaCl, 0.1% NP40, 0.5 mM EDTA, 5% (v/v) glycerol, 1 mM DTT) before overnight incubation with TEV protease at 4 °C. Protein released from the beads was subjected to further purification by calmodulin-affinity chromatography and then dialyzed against storage buffer (10 mM Tris–HCl pH 7.5, 150 mM NaCl, 50% (v/v) glycerol, 1 mM DTT) and stored at −80 °C.

His8-CPD-tagged Sen1 HD (1095–1904) was produced as before^6^. Briefly, Sen1 HD with a cleavable C-terminal His-tag coupled to *Vibriocholerae* MARTX toxin cysteine protease domain was purified from *Escherichia coli* BL21 (DE3) STAR pRARE (Stratagene) cells grown in TB medium. Cells were lysed in buffer containing 20 mM sodium phosphate pH 8.0, 500 mM NaCl, 2 mM MgCl_2_, 30 mM imidazole, 10% (v/v) glycerol, 1 mM β-mercaptoethanol, benzonase, and protease inhibitors, and bound to a Ni^2+^-affinity chromatography column (HisTrap FF, GE Healthcare) followed by on-column tag cleavage using 3C protease^30^. The samples were applied to a HiTrap Heparin HP column (GE Healthcare) equilibrated in buffer A (20 mM Tris–HCl pH 7.5, 200 mM NaCl, 2 mM MgCl_2_, 1 mM DTT) and eluted by developing a gradient to 1 M NaCl. Size-exclusion chromatography was performed using a Superdex HiLoad 200 column (GE Healthcare) equilibrated in GF buffer (20 mM HEPES pH 7.5, 300 mM NaCl, 2 mM MgCl_2_, and 1 mM DTT, 50% (v/v) glycerol) and the purified proteins were stored at −80 °C.

TFIIS was purified as described^24^ via nickel-affinity chromatography. Peak fractions were pooled and diluted five-fold with Mono-S buffer A (50 mM HEPES pH 7.5, 0.01 mM ZnCl_2_, 1 mM DTT, and 10% (v/v) glycerol) and loaded onto a Mono-S anion exchange column (GE Healthcare) equilibrated in Mono-S buffer A. TFIIS was eluted from the Mono-S column by developing a gradient to 1 M NaCl. The second peak fraction was collected and concentrated to ~1 ml (VivaSpin, 3 kDa MWCO, GE Healthcare) and then gel-filtrated (Superdex HiLoad 200 16/60, GE Healthcare) in GF buffer (25 mM HEPES pH 7.5, 250 mM NaCl, 0.01 mM ZnCl_2_, 10 mM DTT, and 10% (v/v) glycerol) before aliquoting and shock-freezing in liquid nitrogen. Single-use aliquots were stored at −80 °C.

Hexahistidine-tagged BirA was purified from 3 l of IPTG-induced BL21 (DE3) culture using Ni-affinity chromatography (His-Trap, GE Healthcare). Briefly, cell pellet was resuspended in lysis buffer (25 mM Tris–HCl pH 8, 250 mM NaCl, 5% (v/v) glycerol) supplemented with EDTA-free Complete Protease Inhibitor (Roche) and lysed using an Avestin C5. After clarification, supernatant was loaded onto 10 ml of nickel-bound metal-chelating resin (HiTrap Chelating column, GE Healthcare). Contaminants were removed by washing resin with 50 mM imidazole, and BirA was eluted by developing an imidazole gradient to 1 M. Protein-containing fractions were pooled and imidazole was removed by buffer exchange (G25 Desalting Column, GE Healthcare) against buffer containing 20 mM Tris–HCl pH 8, 200 mM NaCl, and 5% (v/v/) glycerol. Protein was aliquoted, flash frozen, and stored at −80 °C.

## Supplementary information

Supplementary Information

Reporting Summary

Source Data

## Data Availability

Data supporting the findings of this manuscript are available from the corresponding author upon reasonable request. A reporting summary for this Article is available as a Supplementary Information file. The source data underlying Figs. 1c–f, 3c–e, 4b, d and Supplementary Figs. 1, 2, 3a–f, 4c–f, 5c–f and 8c, d are provided as a Source Data file.

## References

[1] Jin Y, Eser U, Struhl K, Churchman LS (2017). The ground state and evolution of promoter region directionality. Cell.

[2] Ursic D, Himmel KL, Gurley KA, Webb F, Culbertson MR (1997). The yeast SEN1 gene is required for the processing of diverse RNA classes. Nucleic Acids Res..

[3] Hazelbaker DZ, Marquardt S, Wlotzka W, Buratowski S (2013). Kinetic competition between RNA Polymerase II and Sen1-dependent transcription termination. Mol. Cell.

[4] Porrua O, Libri D (2015). Transcription termination and the control of the transcriptome: why, where, and how to stop. Nat. Rev. Mol. Cell Biol..

[5] Han Z, Libri D, Porrua O (2016). Biochemical characterization of the helicase Sen1 provides new insights into the mechanisms of non-coding transcription termination. Nucleic Acids Res..

[6] Leonaite B (2017). Sen1 has unique structural features grafted on the architecture of the Upf1-like helicase family. EMBO J..

[7] Wang MD (1998). Force and velocity measured for single molecules of RNA polymerase. Science.

[8] Revyakin A, Liu CY, Ebright RH, Strick TR (2006). Abortive initiation and productive initiation by RNA polymerase involve DNA scrunching. Science.

[9] Galburt EA (2007). Bactracking determines the force sensitivity of RNAP II in a factor-dependent manner. Nature.

[10] Revyakin A (2012). Transcription initiation by human RNA polymerase II visualized at single-molecule resolution. Genes Dev..

[11] Fazal FM, Meng CA, Murakami K, Kornberg RD, Block SM (2015). Real-time observation of the initiation of RNA polymerase II transcription. Nature.

[12] Palangat M, Larson X, Gnatt A, Block S, Landick R (2012). Efficient reconstitution of transcription elongation complexes for single-molecule studies of eukaryotic RNA polymerase II. Transcription.

[13] Revyakin A, Ebright RH, Strick TR (2004). Promoter unwinding and promoter clearance by RNA polymerase: detection by single-molecule DNA nanomanipulation. Proc. Natl Acad. Sci. USA.

[14] Lerner E (2016). Backtracked and paused transcription initiation intermediate of escherichia coli rna polymerase. Proc. Natl Acad. Sci. USA.

[15] Howan K (2012). Initiation of transcription-coupled repair characterized at single-molecule resolution. Nature.

[16] Graves ET (2015). A dynamic DNA-repair complex observed by correlative single-molecule nanomanipulation and fluorescence. Nat. Struct. Mol. Biol..

[17] Fan J, Leroux-Coyau M, Savery NJ, Strick TR (2016). Reconstruction of bacterial transcription-coupled repair at single-molecule resolution. Nature.

[18] Guo Y, Gralla JD (1998). Promoter opening via a DNA fork junction binding activity. Proc. Natl Acad. Sci. USA.

[19] Murakami KS, Masuda S, Campbell EA, Muzzin O, Darst SA (2002). Structural basis of transcription initiation: an RNA polymerase holoenzyme–DNA complex. Science.

[20] Komissarova N, Becker J, Solter S, Kireeva M, Kashlev M (2002). Shortening of RNA:DNA hybrid in the elongation complex of RNA polymerase is a prerequisite for transcription termination. Mol. Cell.

[21] Larson MH, Greenleaf WJ, Landick R, Block SM (2008). Applied force reveals mechanistic and energetic details of transcription termination. Cell.

[22] Westover KD, Bushnell DA, Kornberg RD (2004). Structural basis of transcription: separation of RNA from DNA by RNA polymerase II. Science.

[23] Bintu L (2011). The elongation rate of RNA polymerase determines the fate of transcribed nucleosomes. Nat. Struct. Mol. Biol..

[24] Kettenberger H, Armache KJ, Cramer P (2003). Architecture of the RNA polymerase II-TFIIS complex and implications for mRNA cleavage. Cell.

[25] Mischo HE (2011). Yeast Sen1 helicase protects the genome from transcription-associated instability. Mol. Cell.

[26] Porrua O, Libri D (2013). A bacterial-like mechanism for transcription termination by the Sen1p helicase in budding yeast. Nat. Struct. Mol. Biol..

[27] Kou SC, Cherayil BJ, Min W, English BP, Xie XS (2005). Single-molecule Michaelis–Menten equations. J. Phys. Chem. B.

[28] Duboc C, Fan J, Graves ET, Strick TR (2017). Preparation of DNA substrates and functionalized glass surfaces for correlative nanomanipulation and colocalization (NanoCOSM) of single molecules. Methods Enzymol..

[29] Feklistov A, Darst SA (2011). Structural basis for promoter -10 element recognition by the bacterial RNA polymerase sigma subunit. Cell.

[30] Youell J, Fordham D, Firman K (2011). Production and single-step purification of EGFP and a biotinylated version of the human rhinovirus 14 3C protease. Protein Expr. Purif..

[31] Izban MG, Luse DS (1992). Factor-stimulated Â¨RNA polymerase II transcribes at physiological elongation rates on naked DNA but very poorly on chromatin templates. J. Biol. Chem..

[32] Veloso A (2014). Rate of elongation by RNA polymerase ii is associated with specific gene features and epigenetic modifications. Genome Res..

