# Supplementary material for: Single-molecule characterization of extrinsic transcription termination by Sen1 helicase
Source: Nat Commun. 2019 Apr 4;10:1545. doi: 10.1038/s41467-019-09560-9 (PMC6449345; doi:10.1038/s41467-019-09560-9)
Supplement: Supplementary file 1 — Supplementary Information [file 41467_2019_9560_MOESM1_ESM.docx]

**SUPPLEMENTARY INFORMATION**

**Single-molecule characterization of extrinsic transcription termination by Sen1 helicase**

Wang et al.

**SUPPLEMENTARY NOTES**

**Single-molecule Michaelis-Menten**

Single-molecule recasting of Michaelis-Menten equation was used to fit the lifetime distributions of Pol II termination by various Sen1 HD concentrations in Fig. 3d.

**(1)**

$$f\left( \tau\right)= \frac{k_{1}^{0}k_{\mathrm{CAT}}}{2a}\left\{ \exp\left[ \left( a+b \right)\tau\right]-\exp[(b -a)\tau] \right\},$$

where

$$a= \sqrt{\frac{1}{4}{(k_{1}^{0}+k_{-1} +k_{\mathrm{CAT}} )}^{2} - k_{1}^{0}k_{\mathrm{CAT}}}, b=-\frac{1}{2}\left( k_{1}^{0}+ k_{-1}+ k_{\mathrm{CAT}} \right), k_{1}^{0}= k_{1}[Sen1 HD]$$

fitting parameters are the rate constants *k*_1_, *k*_-1_ and *k*_CAT_.

**R-loop formation during Pol II transcription**

In addition to the pulses described above, this assay also reveals transcription events initiated from the permanent bubble and which result in R-loop formation (Supp. Fig. 6). R-loops are triple-stranded structures formed when RNA is able to hybridize to its complementary bases on the template strand outside of the context of the transcription bubble. These structures have multiple roles in gene expression and replication for instance [2, 1]. In our assay, R-loop formation is characterized by three regimes: an initial increase in DNA extension when Pol II initiates as before, but which is then followed by an additional increase in DNA extension (to the maximal DNA extension observed in the absence of supercoiling), and then finally a regime in which DNA extension decreases rapidly and linearly with time, dramatically reducing the extension of the DNA. In this regime the DNA extension decreases at a rate of ~50 nm/s. This occurs in 78 out of 485 transcription pulses (i.e. a probability of 0.16 ± 0.02, SD) when a dinucleotide is used to prime transcription from the permanent bubble, but in 83 out of 102 transcription pulses (i.e. a probability of 0.81 ± 0.09, SD) when a 9-mer RNA is used instead (see Supp. Fig. 6). No such effect was observed when the dinucleotide was used to prime transcription from a positively supercoiled template (0 occurrences out of 350 transcription pulses). Because this category of events is also abolished if RNase H is included in the experiment with dinucleotide primer (0 occurrences out of 227 transcription events), we conclude that it reflects the formation of an R-loop in the DNA.

We propose that the additional increase and subsequent decrease in DNA extension is due to topological coupling between Pol II elongation and formation of positive supercoils in the DNA, which can occur if a closed topological domain is formed between the permanent bubble and Pol II (see Supp. Fig. 6). Such a closed topological domain could be generated if RNA remains annealed at the bubble rather than engaging in the RNA exit channel of Pol II, forming a hybrid as Pol II advances, or alternatively if the RNA invades the template DNA between the permanent bubble and Pol II. In this model the rate of decrease of DNA extension in this regime (~50 nm/s) is a reflection of the velocity of Pol II (~10 bp/s) and the fact that by extending an R-loop by 10 bases the DNA must topologically compensate untwisting of DNA within the closed topological domain by forming a positive supercoil (50 nm/turn, Supp. Fig. 2) outside of the closed topological domain. This causes the DNA to contract by 50 nm in approximately 1 s. In conclusion, our system is suitable to study how intrinsic and extrinsic factors can influence or regulate the formation of R-loops.

**Construct sequences:**

Pol2-144-444-his

5’-GGTACCACGCGTttacgccagctggcgaaaggCCTAGGgggatgtgctgcaaggcgatAAAAAAAAAGCCCCCGGAAGATGCATCTTCCGGGGGCTTTCtaagttgggtaacgccagggACGCGTttttcccagtcacgacgttgtaaaacgacggccagtgaatccgtaatcatggtcatACTAGTGTTCCGAGTGCAGTTCGCAAGCTTtagcgaagaggcccgcaccgatcgcccttcccaacagttgcgcagcctgaatggcgaatggcgctttgcctggtttccggcaccagaagcggtgccggaaagctggctggagtgcgatcttcctgaggccgatactgtcgtcgtcccctcaaactggcagatgcacggttacgatgcgcccatctacaccaacgtgacctatcccattacggtcaatccgccgtttgttcccacggagaatccgacgggttgttactcgctcacatttaatgttgatgaaagctggctacaggaaggccagacgcgaattatttttgatggcgttaactcggcgtttcatctgtggtgcaacgggcACGCGTgctgggtcggttacggccagGAAAGCCCCCGGAAGATGCATCTTCCGGGGGCTTTTTTTTTgacagtcgtttgccgtctgaGCTAGCatttgacctgagcgcattttACGCGTGGTACC

Pol2-444-444-his

5’-GGTACCttacgccagctggcgaaaggGCGGCCGCgggatgtgctgcaaggcgatAAAAAAAAAGCCCCCGGAAGATGCATCTTCCGGGGGCTTTCtagcgaagaggcccgcaccg**CCTAGG**atcgcccttcccaacagttgcgcagcctgaatggcgaatggcgctttgcctggtttccggcaccagaagcggtgccggaaagctggctggagtgcgatcttcctgaggccgatactgtcgtcgtcccctcaaactggcagatgcacggttacgatgcgcccatctacaccaacgtgacctatcccattacggtcaatccgccgtttgttcccacggagaatccgacgggttgttactcgctcacatttaatgttgatgaaagctggctacaggaaggccagacgcgaattatttttgatggcgttaactcggcgtttcatctgtggtgcaacgggcgctgggtcggttacggccagACTAGTGTTCCGAGTGCAGTTCGCAAGCTTtagcgaagaggcccgcaccgatcgcccttcccaacagttgcgcagcctgaatggcgaatggcgctttgcctggtttccggcaccagaagcggtgccggaaagctggctggagtgcgatcttcctgaggccgatactgtcgtcgtcccctcaaactggcagatgcacggttacgatgcgcccatctacaccaacgtgacctatcccattacggtcaatccgccgtttgttcccacggagaatccgacgggttgttactcgctcacatttaatgttgatgaaagctggctacaggaaggccagacgcgaattatttttgatggcgttaactcggcgtttcatctgtggtgcaacgggc**ACGCGT**gctgggtcggttacggccagGAAAGCCCCCGGAAGATGCATCTTCCGGGGGCTTTTTTTTTgacagtcgtttgccgtctgaGCTAGCatttgacctgagcgcattttGGTACC

Pol2-G-less-cassette

5’-GGTACCAAGCTTGCGAACTGCACTCGGAACGAATTCATACACATCAAACATACACCCAACTAACCAACTACTAACACACAATCACACACTCACCTACCATAACCAACTCACAACAACAACACCTCTCCATACTCTACTCCTATACCACACTAGTATGACCATGATTACGGATTCACTGGCCGTCGTTTTACAACGTCGTGACTGGGAAAA**ACGCGT**GGTACC

T5N25-178his

5’-GGTACCAAGCTAAAAATTTATTTGCTTTCAGGAAAATTTTTCTGTATAATAGATTCATAAATTTGAGAGAGGAGTCACTAGAAGCTTGCGAACTGCACTCGGAACACTAGTATGACCATGATTACGGATTCACTGGCCGTCGTTTTACAACGTCGTGACTGGGAAAAACGCGTCCCTGGCGTTACCCAACTTAGAAAGCCCCCGGAAGATGCATCTTCCGGGGGCTTTTTTTTTATCGCCTTGCAGCACATCCCCCTAGGCCTTTCGCCAGCTGGCGTAAACGCGTGGTACC

**SUPPLEMENTARY REFERENCES**

[1] J.M. Santos-Pereira and A. Aguilera. R loops: new modulators of genome dynamics and function. *Nat. Rev. Genetics*, 16:583–597, 2015.

[2] K. Skourti-Stathaki and N.J. Proudfoot. A double-edged sword: R-loops as threats to genome integrity and powerful regulators of gene expression. *Genes Dev.*, 28:1384–1396, 2014.

**SUPPLEMENTARY FIGURES**


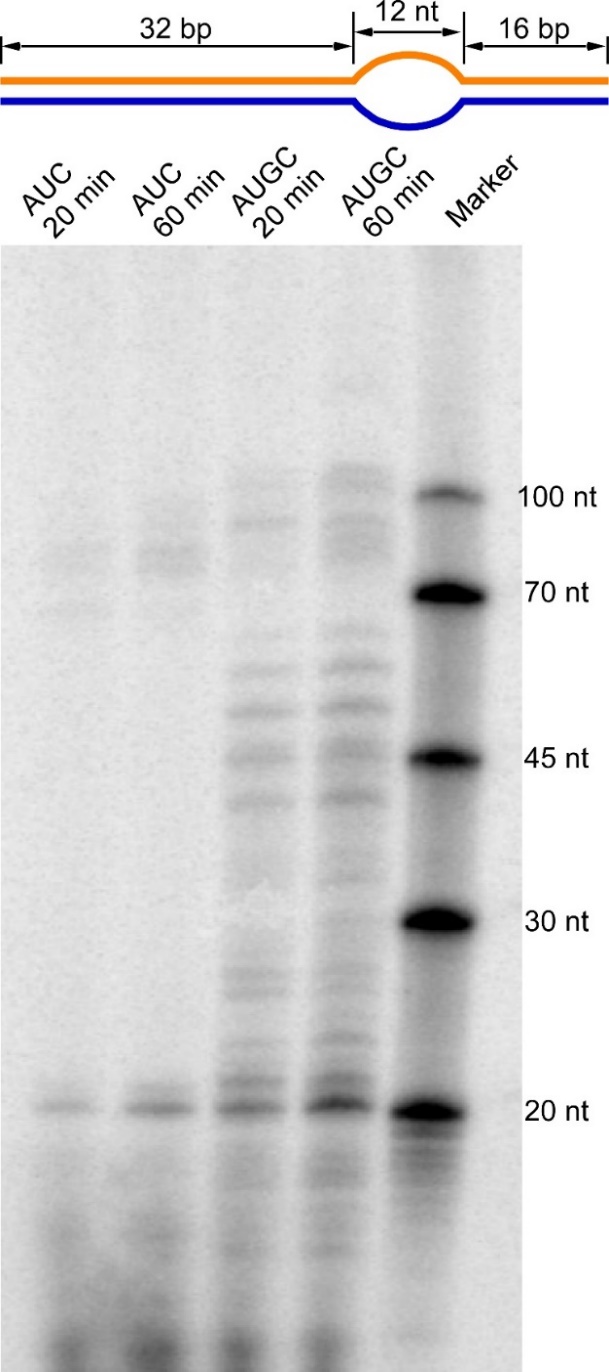


**Supplementary Figure 1. Bulk analysis of transcription from the bubble construct. Top,** Sketch of the substrate. The 12 nt permanent bubble sequence and the flanking 16 and 32 bp sequences are identical to those used in the single-molecule assay. **Bottom**, Transcription run-off assays carried out in the presence of four nucleotides and an initiating dinucleotide (GpA, see Materials & Methods) show that the scaffold sustains bidirectional transcription in these conditions. In the presence of only 3 NTPs generation of longer transcripts from the 32 bp segment is restricted, demonstrating that transcription is therefore oriented. Source data are provided as a Source Data file.


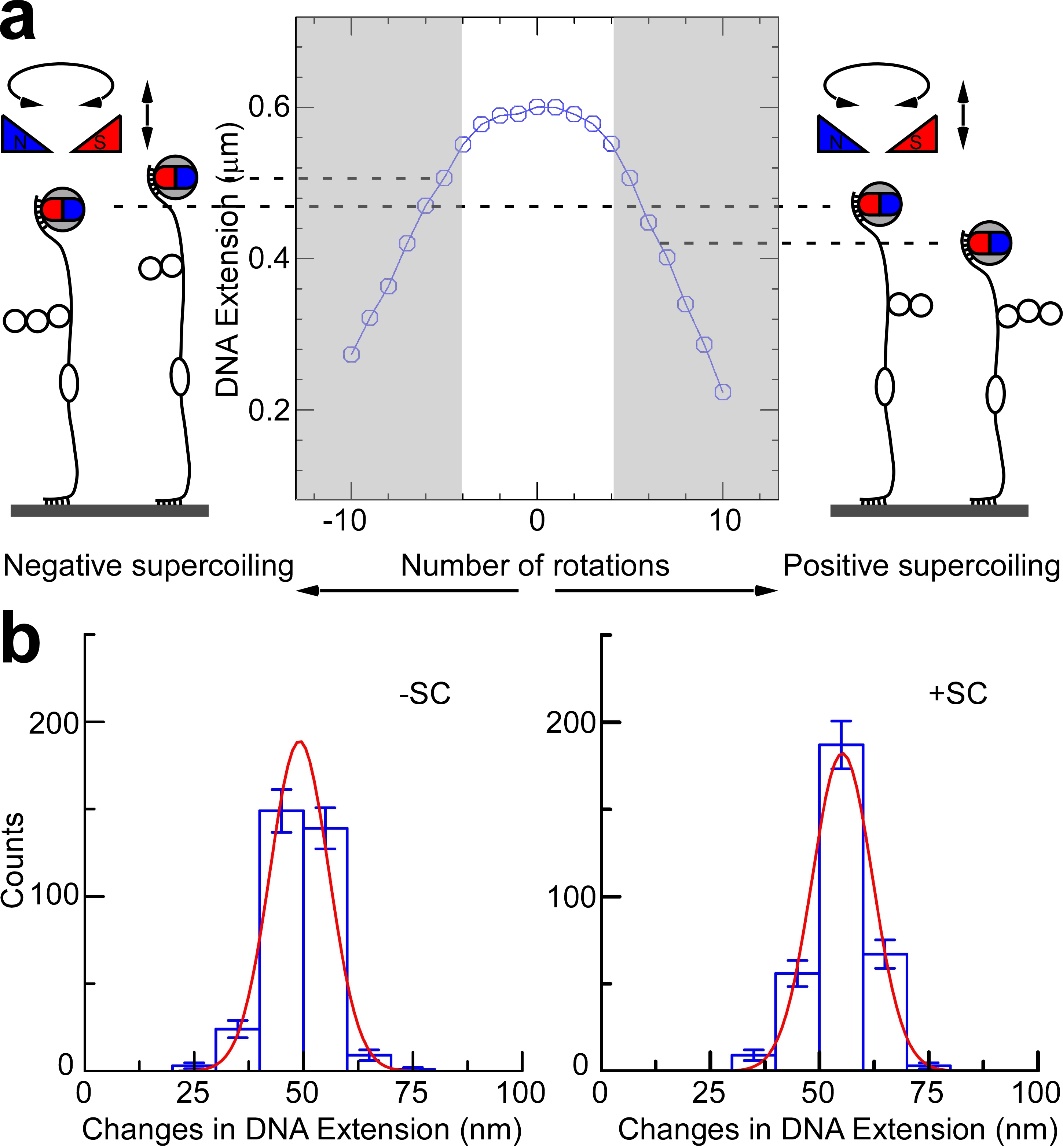


**Supplementary Figure 2. Topological calibration of bubble-containing DNA. a,** Experimental extension vs. supercoiling curve for bubble-containing DNA extended by a constant force (F = 0.3 pN). A slope can be determined in the linear regime (grey zones) of the extension vs. supercoiling cuve. **b,** Linear slopes of extension vs. supercoiling curves for (left panel) negative supercoiling and (right panel) positive supercoiling are fit to a Gaussian distribution (red lines), respectively yielding values of 49 ± 7 nm (SD, n=325) and 55 ± 7 nm (SD, n=322). Source data are provided as a Source Data file.


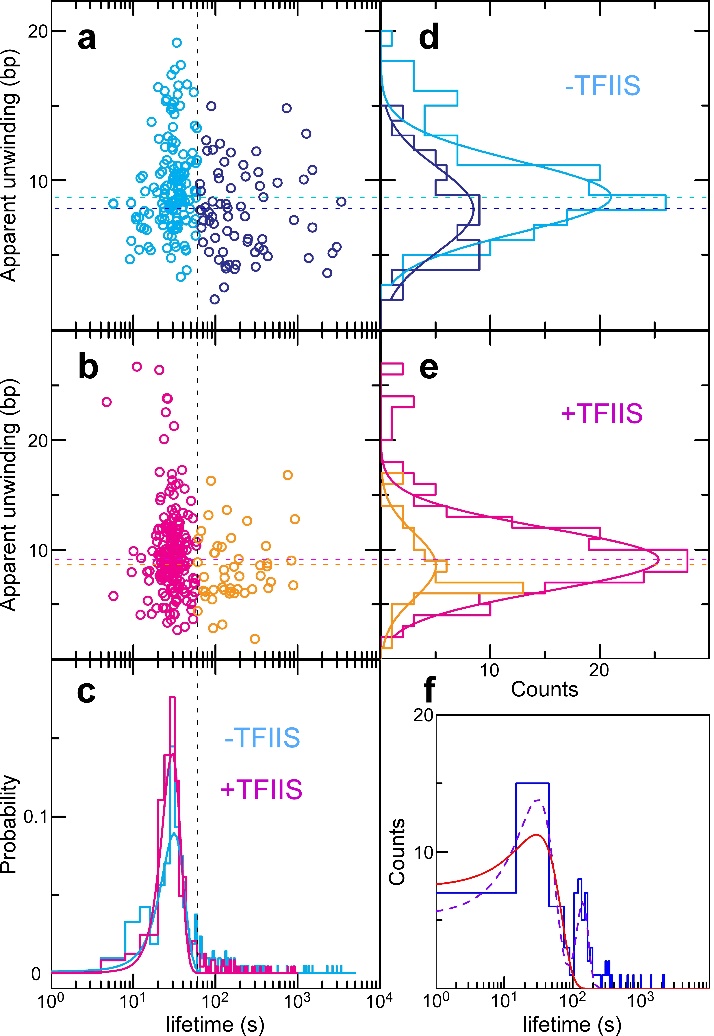


**Supplementary Figure 3. Observation of TFIIS activity.** We present 2-D plots of transcription pulses obtained on the Pol2-444-444-his construct, for which two 444 bp transcripts with different sequences can be produced bidirectionally from the permanent bubble, **a,** in the absence or **b,** in the presence of 25 nM TFIIS. **c,** The 2-D plots are projected along the lifetime axis and clearly show a dominant peak reflecting a Gaussian distribution of pulse durations. In the absence of TFIIS the peak is located at 31.4 ± 1.0 s (SEM) (SD = 10.1 s, n =214 events total), while in the presence of TFIIS the peak is located at 29.8 ± 0.6 s (SEM) (SD = 7.8 s, n =244 events total). In the absence of TFIIS, 68 out of 214 transcription pulses (i.e. 31.8 ± 3.9 % of events) take significantly longer than the average pulse, whereas in the presence of TFIIS only 47 out of 244 transcription pulses (i.e. 19.3 ± 2.8 % of events). **d,e,** Based on the temporal analysis we next separate Pol 2 molecules into two populations (normal in cyan or magenta; slow in dark blue and orange) for further amplitude analysis. The amplitude distributions are fit to Gaussians, giving: (light blue) 8.9 ± 0.2 bp (SEM, n = 146); (blue) 8.0 ± 0.4 bp (SEM, n = 68); (magenta) 9.1 ± 0.2 bp (SEM, n = 197); (yellow) 8.5 ± 0.6 bp (SEM, n = 47). **f,** Pulse duration observed upon replacing the *his* terminator by the *tR2* terminator is fit by a single Gaussian (solid red line, maximum at 30.0 ± 8.6 s, n = 86). A double Gaussian function (dashed violet line, maximums at 31.4 ± 4.3 and 140.4 ± 5.7, n=86) better captures a secondary peak absent in experiments with *his* terminator and thus likely corresponding to Pol II pausing at the *tR2* terminator. Source data are provided as a Source Data file.


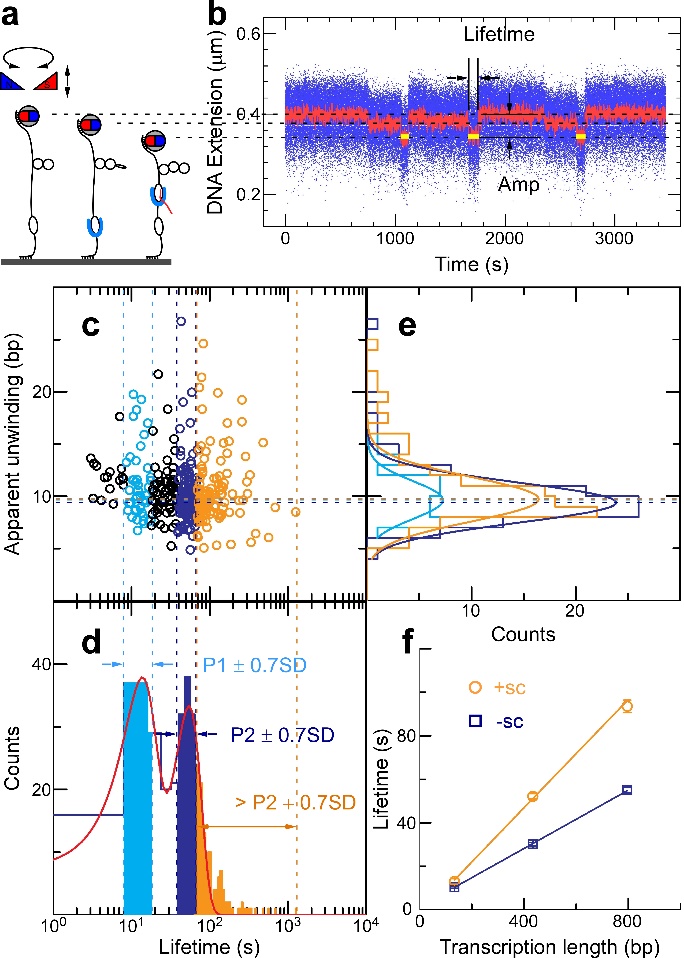


**Supplementary Figure 4. Observation of transcription pulses on positively supercoiled DNA. a,** Sketch of the system showing how the extension decreases upon formation of a transcription bubble on a positively supercoiled substrate. We note that positive supercoiling introduces a new detectable state in the system in which the DNA extension decreases slightly from the baseline just prior to bonafide transcription. This likely results from torsional rearrangement of the unpaired bubble. **b,** Time-trace obtained on positively supercoiled DNA (σ = 0.015) showing a succession of transcription pulses (highlighted in yellow) which can be characterized by both their duration and their amplitude. **c,** Two-dimensional representation of each transcription pulse as a point characterized by (Δl, t) co-ordinates. **d,** Temporal projection of the 2D plot can be fit (red line) to a double Gaussian function, giving two peaks located at 13.0 ± 1.3 s (SEM) and 54.5 ± 2.6 s (SEM) (n =350 events total). Color code gives data ranges used to specify distinct populations. (Cyan) Pol II molecules transcribing the short 144 bp transcript; (Navy blue) Pol II molecules transcribing the longer 444 bp transcript; (Orange) Pol II molecules displaying abnormally long elongation times. **e,** Spatial projections of the 2D plot based on the peaks identified and color-coded as in (D) are fit to Gaussian distributions, yielding transcription bubble sizes of: (Cyan) 9.6 ± 0.3 s (SEM, n = 45) (Navy blue) 9.4 ± 0.2 s (SEM, n = 122) and (Orange) 9.7 ± 0.2 s (SEM, n = 106). TFIIS was present at 25 nM in this assay. **f,** Mean pulse duration (t) as a function of transcript unit length for positively supercoiled DNA. Data were obtained from Peak 1 for the Pol2-144-444-his construct and the single peaks obtained for the Pol2-444-444 and Pol2-806-806-his constructs. Fitting to a linear model t = L/v + t_0_ gives a velocity v = 8.0 ± 0.3 bp/s and an intercept t_0_ = -3.0 ± 1.7 s. Data for negatively supercoiled DNA are reproduced from Fig. 1 for the sake of comparison. Error bars represent SEM. Source data are provided as a Source Data file.


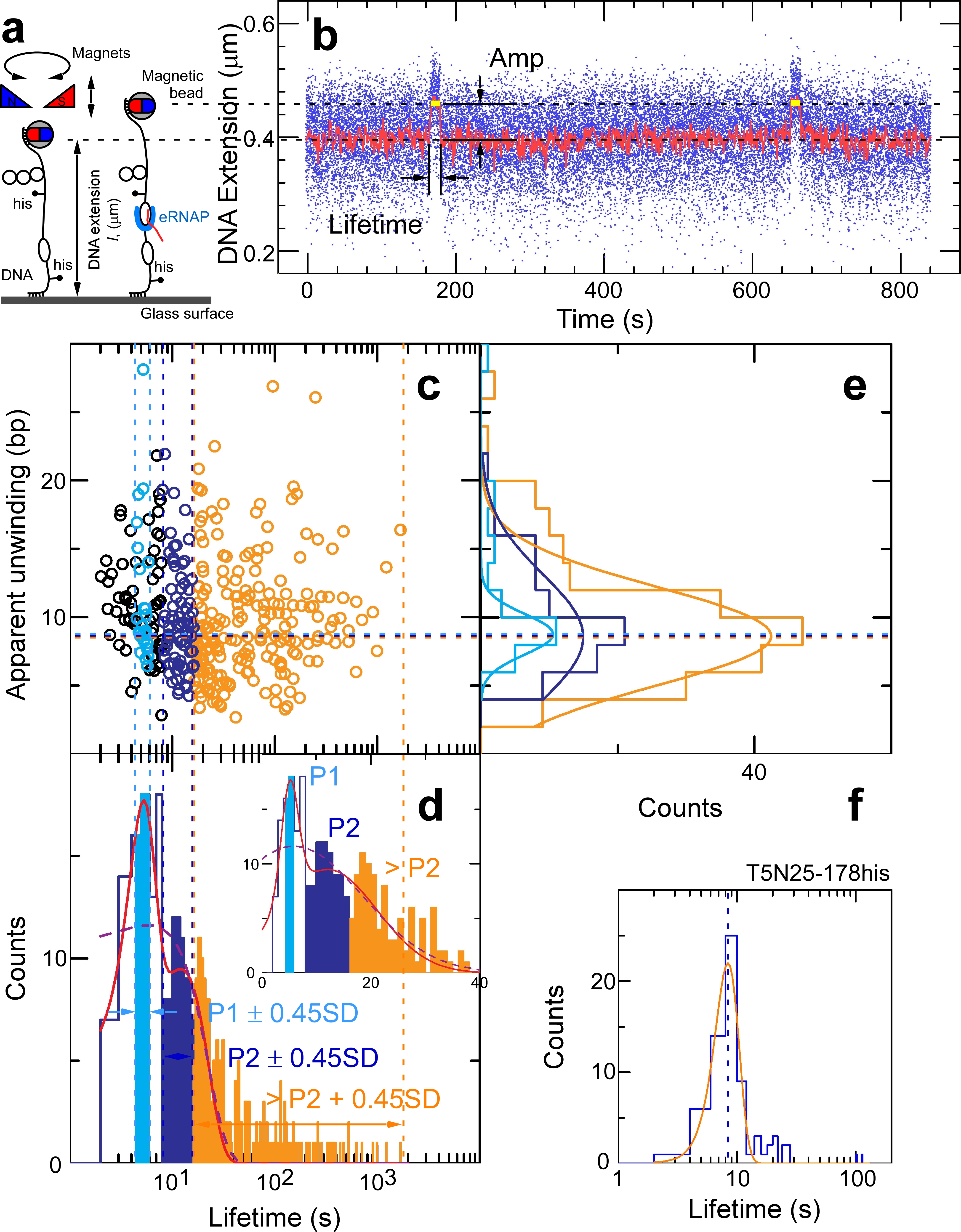


**Supplementary Figure 5. Transcription pulses observation of *E. Coli* RNAP. a**, Sketch of the system showing the generation of transcription pulses upon formation of a transcription bubble inside of *ec*RNAP. **b,** Time-trace of *ec*RNAP transcription on the Pol2-144-444his construct under negative supercoiling (σ = 0.027) showing a succession of transcription pulses (highlighted in yellow) whose duration and amplitude can well characterized. **c,** Two-dimensional representation of each transcription pulse as a point characterized by (Δl,t) coordinates. **d,** Temporal projection of the 2-D plot (scaled in log) can be better fit to a double-Gaussian (red line) with a reduced chi-square of 0.60 than to a single-Gaussian (violet dash line) with a reduced chi-square of 0.94. Double-Gaussian fit gives two peaks located at 5.2 ± 0.5 s (SEM) and 12.1 ± 4.5 s (SEM) (n = 373 events total). Color code was used to specify distinct transcription populations. (Cyan) *ec*RNAP transcribing the 144 bp transcript; (Navy blue) *ec*RNAP transcribing the 444 bp transcript; (Orange) *ec*RNAP transcribing for an abnormally long time. Insert is the temporal projection in linear scale from 0 to 40 s. **e,** Spatial projections of the 2-D plot based on the peaks identified and color-coded in (d) are fit to single-Gaussian distributions, yielding transcription bubble sizes of: (Cyan) 8.8 ± 0.4 bp (SEM, n = 26) (Navy blue) 8.6 ± 0.9 bp (SEM, n = 78) and (Orange) 8.6 ± 0.3 bp (SEM, n = 209). **f,** Distribution of *ec*RNAP transcription elongation time on the T5N25-178his construct under negative supercoiling (σ = 0.029). Single-Gaussian fit gives the peak position of 8.4 ± 0.3 s (SEM, n = 71) and the transcription rate can be obtained as 21.2 ± 0.7 bp/s considering the transcription length of 178 bp. Source data are provided as a Source Data file.


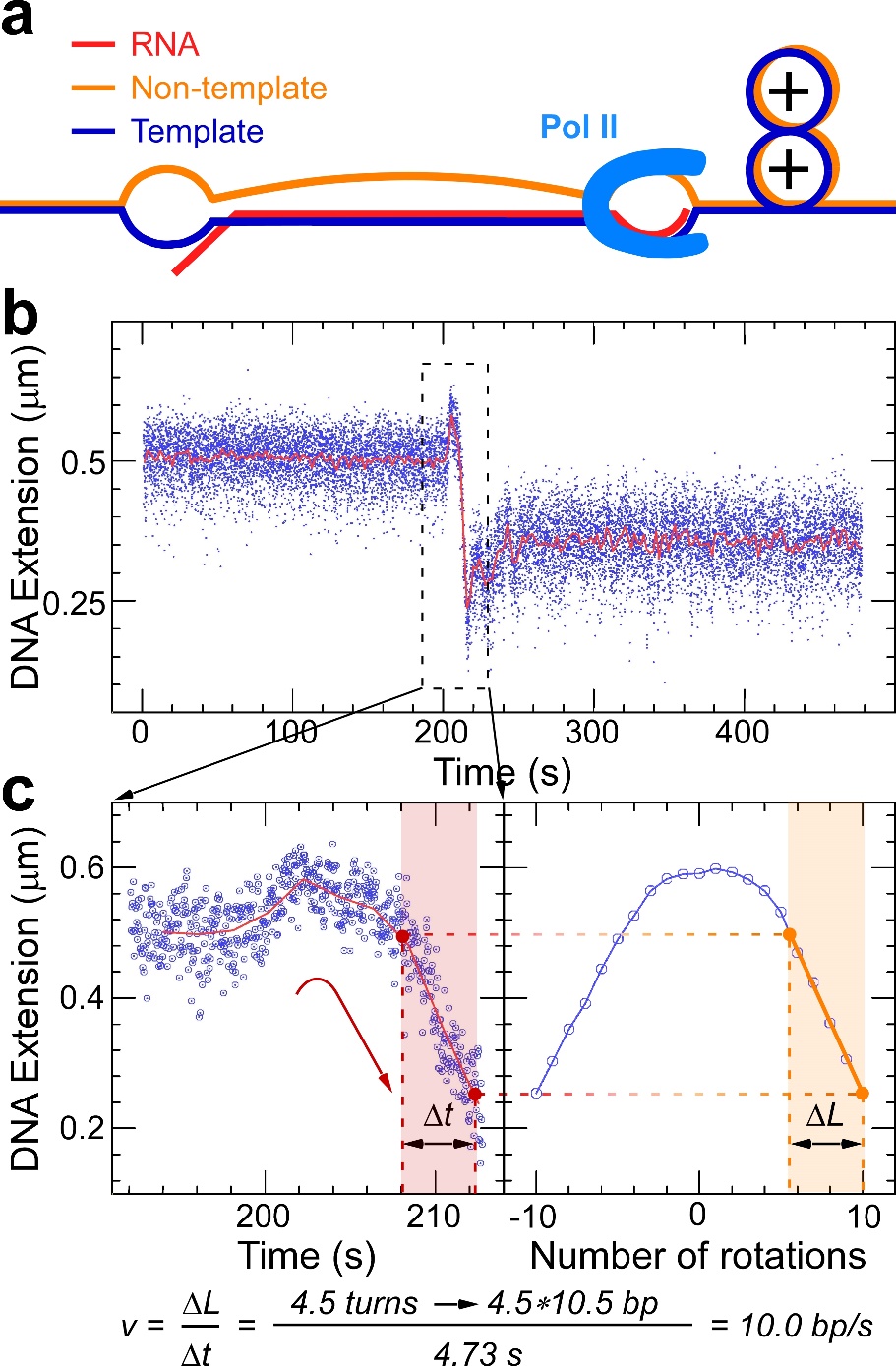


**Supplementary Figure 6.** **Real-time detection of R-loop formation by bubble-initiated Pol 2. a,** Schematic representation of model for R-loop formation and induction of compensatory positive supercoils. **b,** Time-trace obtained on negatively supercoiled DNA showing an initial increase in DNA extension as Pol 2 initiates transcription, but which is rapidly followed by a quick decrease in DNA extension. **c,** (Left panel) Expanded view of the transcription pulse shown in (a) with (right panel) comparison to an extension vs. supercoiling curve. The quick decrease in DNA extension is characterized by the change in extension, ΔL, and the time in which it occurs, Δt. If we hypothesize that the change in extension is due to topological formation of plectonemic supercoils, then we conclude that the plectonemic supercoils appear at a rate of approximately 1 supercoil/second. This is consistent with the measured velocity of Pol 2 in this assay (10 bases/second). Plectonemic supercoils could be formed in front of the Pol 2 if there is a compensatory, negatively supercoiled domain behind the Pol 2.


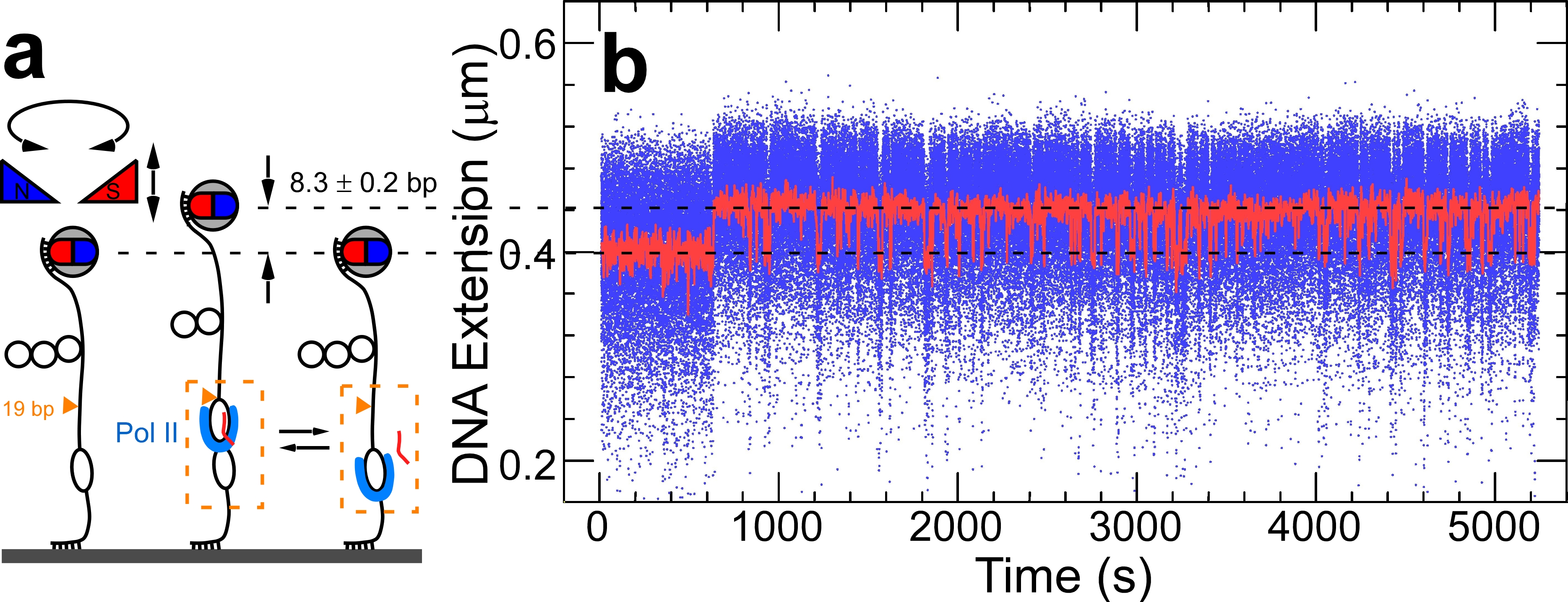


**Supplementary Figure 7.**  **Pol2 stalled at +19 does not form a stable TEC. a,** Sketch of the experiment depicting how Pol 2 stalled at +19 can return to its initial position on the bubble from which it initiated. **b,** Time-trace obtained on negatively supercoiled DNA showing an initial increase in DNA extension, followed by numerous transient decreases in DNA extension. These decreases are consistent with a phenomenon in which the stalled Pol 2 shuttles back and forth between its initial position on the bubble and the +19 position, causing the blebbed bubble to fluctuate in and out of existence.


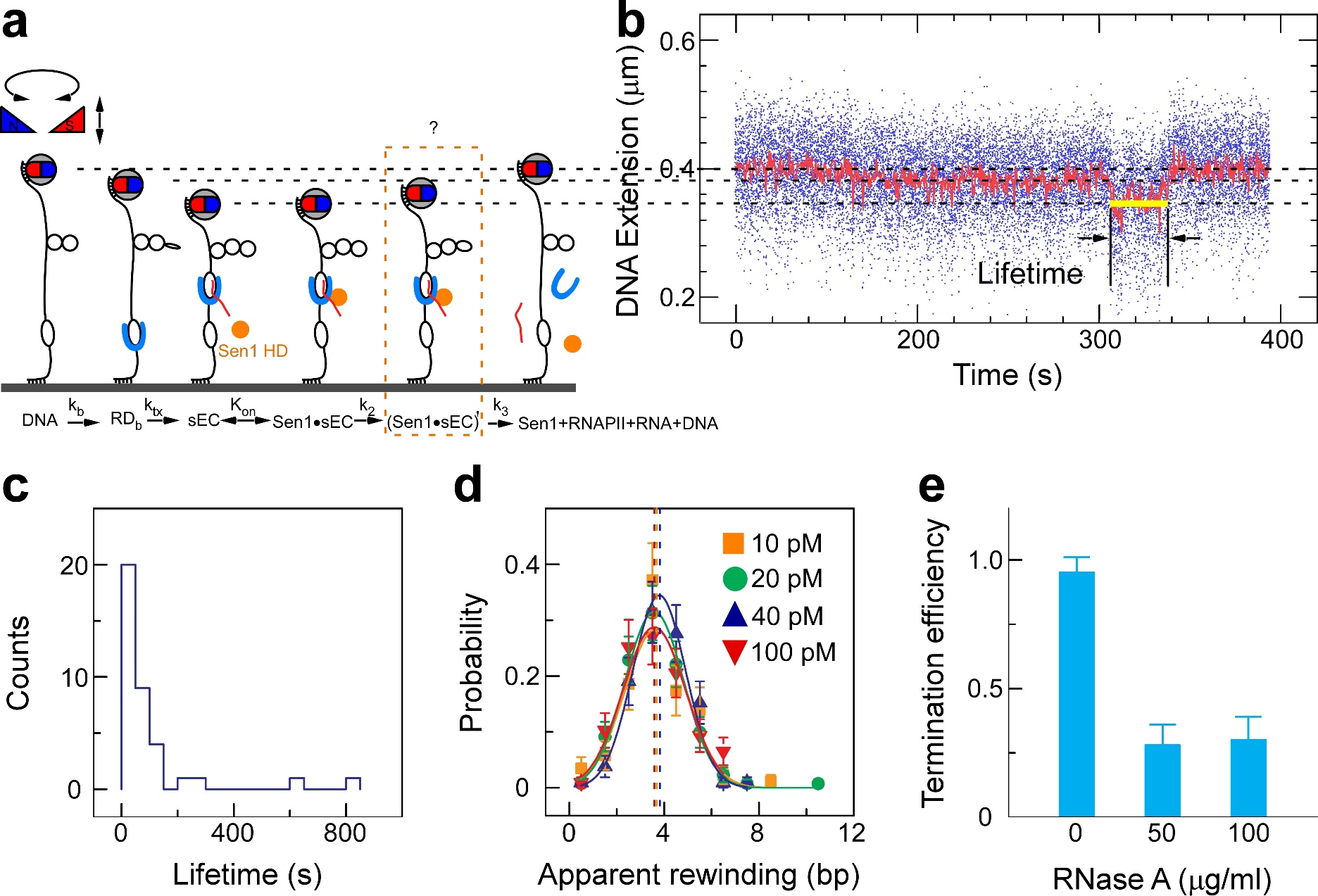


**Supplementary Figure 8.**  **Sen1 HD rapidly displaces stalled Pol 2 from positively supercoiled DNA. a,** Sketch of the experiment. For positively supercoiled DNA transcription is observed as a decrease in DNA extension (see Supp. Fig. 4). **b,** Time-trace showing displacement of stalled Pol 2 by Sen1 HD on positively supercoiled DNA. Both the amplitude of unwinding in the stalled elongation complex, and the lifetime of the stalled elongation complex, can be determined. No intermediate can be observed during termination on a positively supercoiled DNA substrate. **c,** Lifetime distribution of stalled elongation complex on positively supercoiled DNA in the presence of 40 pM Sen1 HD. The mean lifetime obtained is 99.7 ± 27.1 (SEM, n = 37). **d,** The apparent rewinding of the transcription bubble upon formation of the Sen1-Pol2 intermediate on negatively supercoiled DNA is independent of Sen1 concentration and measures approximately 4 bases (orange curve: 3.6 ± 0.2 bp (SEM, n = 86); green curve: 3.6 ± 0.1 bp (SEM, n =131); blue curve: 3.8 ± 0.1 bp (SEM, n =105); red curve: 3.6 ± 0.1 bp (SEM, n=107)). **e**, the efficiency of Pol II termination by Sen1 HD is reduced in the presence of RNase A. Source data are provided as a Source Data file.

**Primer list 5’-3’**

| RPOC_F | GAGAGATCTAGAGACCTTCTGGATCTCGTCCACCAGG |
| --- | --- |
| RPOC_R | GAGAGACCTGCAGGACATCAAGGACGAGGTGTGG |
| 444_LF | ggccCCTAGGatcgcccttcccaacagttgc |
| 444_LR | ggACTAGTctggccgtaaccgacccagc |
| 444_RF | ggttGGCGCGCCagcgaagaggcccgcacc |
| 444_RR | ggcgACGCGTgcccgttgcaccacagatg |
| Non-tem1 | AGCTGGATACTTACAGCCATTACAGTTACGCCTACTCCATTCCATCCC |
| Tem1 | CTAGGGGATGGAATGGAGTATTCGCCGTGTCCATGGCTGTAAGTATCC |
| Tem2 | AATTGGGATGGAATGGAGTATTCGCCGTGTCCATGGCTGTAAGTATCC |
| Non-tem3 | CTCCAGGTACCAAGCTGGATACTTACAGCCATTACAGTTACGCCTACTCCATTCCATCCC |
| Tem3 | GGGATGGAATGGAGTATTCGCCGTGTCCATGGCTGTAAGTATCCAGCTTGGTACCTGGAG |

Non-tem1, tem1 and tem2 were 5’ phosphorylated.
